# Decision pathways for genetically modified phosphate-solubilizing bacteria: a four-dimensional synergistic decision-making framework from genetic targets to field deployment

**DOI:** 10.3389/fpls.2026.1949787

**Published:** 2026-10-28

**Authors:** Bo Chen, Yan Zhang, Chengyi Zou, Caiming Gou, Jia Li

**Affiliations:** 1 School of Agriculture, Forestry and Food Engineering, Yibin University, Yibin, China; 2 Solid-State Fermentation Resource Utilization Key Laboratory of Sichuan Province, Yibin University, Yibin, China

**Keywords:** adaptive laboratory evolution, biocontainment, evolutionary escape, evolutionary stability, four-dimensional reciprocal feedback framework, genetic engineering, phosphate-solubilizing bacteria, twelve feedback loops

## Abstract

Genetically modified phosphate-solubilizing bacteria (PSB) lack systematic feedback integration among four dimensions — gene-target selection(D1), engineering-strategy design (D2), field performance (D3) and environmental-safety constraints (D4) — giving rise to a fundamental dilemma across the research field. The key contributions of this Perspective is that we propose the Four-Dimensional Reciprocal Feedback Framework (4D-RFF), which places these four dimensions on a unified analytical plane and delineates how twelve categories of bidirectional feedback interactions reshape the decision logic governing the translation of engineered strains from bench to field. The central thesis is that the field’s current bottleneck is not a technical deficiency in any single dimension, but rather a disconnect between dimensions: misalignment between target identification and engineering strategy, between engineering design and agronomic performance, and between safety assessment and the full product development pipeline. This disconnect, we argue, is the underlying reason why engineered PSB strains rarely progress to commercial deployment. We further contend that suboptimal field performance is not a terminal outcome but a critical feedback signal that should inform iterative design refinement. However, traits selected via adaptive laboratory evolution (ALE) under homogeneous liquid culture conditions do not necessarily confer fitness advantages in heterogeneous field soils; ALE pre-adaptation should therefore be conducted in soil mi exudates. Environmental safety, similarly, should not be treated as an afterthought appended crocosms amended with target soil matrices and root late in development. Instead, evolutionary escape frequency can be driven below detection limits through multiple redundant orthogonal dependency (MROD) design of essential genes—although quantitative prediction of escape rates still relies on several strong assumptions that require calibration against deep mutational scanning data. We conclude by calling for a shift from single-gene manipulation toward systems-level design in PSB engineering, and identify evolutionary stability engineering and quantitative predictive modeling of escape frequency as two pivotal scientific challenges for the next five years.

## 1 Introduction

### 1.1 Current status and mitigation strategies of the phosphorus crisis

Phosphorus is an essential macronutrient for plant growth and development, participating in core biological processes including nucleic acid synthesis, energy metabolism, membrane integrity maintenance and signal transduction (Michigami et al., 2018). Global agricultural production relies heavily on phosphate fertilizers, whose demand continues to rise at an annual rate of approximately 2.5%. Nevertheless, phosphate rock is a non-renewable resource, and high-grade phosphate rock reserves are being depleted rapidly. Some studies predict that economically mineable phosphate rock reserves may be exhausted within the next 50–100 years (Timofeeva et al., 2022). Meanwhile, the seasonal utilization efficiency of chemical phosphate fertilizers applied to soil is merely 10%–25%. Most remaining phosphorus is immobilized by soil minerals into insoluble forms such as iron phosphate, aluminum phosphate and calcium phosphate, or lost via surface runoff, triggering aquatic eutrophication (Fatima et al., 2021). Soils generally contain substantial total phosphorus, yet bioavailable phosphorus often accounts for less than 1% of total soil phosphorus (Wang et al., 2023). Soil phosphorus exists in organic and inorganic chemical forms. Organic phosphorus accounts for 30%–80% of total phosphorus, whereas inorganic phosphorus exists predominantly as insoluble bound fractions (Qin et al., 2020; Ducousso-Détrez et al., 2022). China’s “Zero Growth of Chemical Fertilizers” policy imposes rigid requirements for biological phosphorus replacement technologies, further highlighting the urgency of developing alternatives to mineral phosphate fertilizers (Bohua et al., 2023). Therefore, converting immobilized, non-bioavailable soil phosphorus into plant-accessible forms represents a critical scientific challenge to alleviate the phosphorus crisis. Phosphate-solubilizing bacteria (PSB) convert insoluble phosphorus into plant-available forms by secreting organic acids and phosphatases, offering a promising biological strategy to tackle the soil phosphorus crisis (Hayat et al., 2010; Behera et al., 2013).

### 1.2 The promise and practical gaps of phosphate-solubilizing bacteria

Phosphate-solubilizing bacteria (PSB) are a group of bacteria capable of transforming insoluble inorganic or organic phosphorus in soil into plant-available forms. Their phosphorus-solubilizing mechanisms fall into two major categories. First, inorganic phosphorus solubilization: bacteria secrete organic acids such as gluconic acid and 2-ketogluconic acid to chelate metal cations and release phosphate anions (Hsu et al., 2015; Rasul et al., 2019). Second, organic phosphorus mineralization: microbes produce hydrolytic enzymes including acid phosphatase, alkaline phosphatase and phytase to decompose organic phosphorus compounds into inorganic phosphate (Hayat et al., 2010; Neal et al., 2017). In addition, some PSB can indirectly promote plant growth by producing siderophores, exopolysaccharides and phytohormones (Saeed et al., 2021; Santoyo et al., 2021).

Since the microbial phosphate-solubilizing phenomenon was first discovered in the early 20th century, numerous studies have isolated phosphate-solubilizing bacterial taxa from diverse soil types and plant rhizospheres, including genera such as *Pseudomonas*, *Bacillus*, *Burkholderia*, *Enterobacter*, *Serratia*, and *Pantoea* (Zhao et al., 2013; You et al., 2019; Ribeiro et al., 2020). In extreme environments such as low-temperature soils, psychrophilic and cold-tolerant PSB also exhibit phosphate-solubilizing capacity, expanding the ecological scope of PSB application (Rizvi et al., 2021). Greenhouse and pot experiments have demonstrated that PSB inoculation can significantly increase crop biomass, phosphorus uptake and grain yield (Bakhshandeh et al., 2014; Anzuay et al., 2017).

Nevertheless, translating PSB inoculants from laboratory research to large-scale field deployment remains challenging. The field performance of PSB is highly variable: the efficacy of a single strain can vary by several folds across soil types, crop species and growing seasons (Gutiérrez et al., 2022; Poppeliers et al., 2023). Most engineered strains and PSB formulations fail to stably colonize target rhizospheres, and their functional expression declines markedly within weeks after inoculation (Romano et al., 2020). Commercially available PSB products are limited in diversity and lack validation for adaptation to specific soil–crop systems (Domínguez-González et al., 2022; Wang et al., 2023). This dilemma — robust yield benefits observed in controlled experiments but poor prospects for commercialization — motivates the core research question addressed in this Perspective.

### 1.3 Limitations of existing reviews and proposition of the present framework

Numerous reviews have covered various facets of PSB research from distinct perspectives. Some reviews focus on taxonomic descriptions of phosphate-solubilizing mechanisms (Behera et al., 2013; Shaw et al., 2022); others summarize plant-growth-promoting performance and inoculation techniques with an application-oriented emphasis (Fatima et al., 2021; Timofeeva et al., 2022); additional reviews address molecular underpinnings and key regulatory networks (Kaur et al., 2021). In recent years, a small number of reviews have begun to touch upon the prospects of synthetic biology tools for PSB engineering (Wang et al., 2023), alongside design strategies for synthetic microbial communities in sustainable agriculture (Liang et al., 2022; Shayanthan et al., 2022).

Nevertheless, existing reviews suffer from a fundamental limitation: they treat genetic targets, engineering strategies, application performance, and environmental safety as four independent research dimensions discussed in isolation, failing to uncover the interactive relationships among these components. In practical research practice, the selection of engineering strategies is constrained by the molecular properties of targets — single-gene targets are amenable to overexpression strategies, whereas multi-gene pathways require consideration of heterologous expression or genomic integration (Elena et al., 2014). Field application performance, in turn, shapes target selection: strains with poor rhizosphere colonization capacity cannot deliver practical benefits even if they possess robust phosphate-solubilizing activity (Romano et al., 2020). Environmental safety considerations should be incorporated from the stage of target selection, rather than appended as an afterthought once engineered strains have been constructed (Hoffmann et al., 2023). Furthermore, cutting-edge advances in synthetic biology concerning biocontainment and genetic isolation (Jia et al., 2017; Stirling et al., 2017), as well as quantitative modeling efforts for evolutionary escape (Bull and Barrick, 2017; Arbel-Groissman et al., 2023), have not yet been integrated into discussions on PSB genetic engineering. Notably, feedback relationships between genetic targets (D1) and application performance (D3), as well as between genetic targets (D1) and environmental safety (D4), are almost entirely absent from existing literature, representing a profound knowledge gap that extends beyond mere insufficient discussion.

Herein, we propose the Four-Dimensional Reciprocal Feedback Framework (4D-RFF), which places four dimensions — genetic targets (D1), engineering strategies (D2), application performance (D3), and environmental safety (D4) — on a unified analytical plane, and systematically demonstrates twelve categories of bidirectional reciprocal feedback relationships among them (F1–F12, where F = Feedback, denoting feedback loops). Genetic targets (D1) encompass essential genes, operon systems, and regulatory networks. Engineering strategies (D2) include CRISPR gene editing, overexpression and promoter engineering, and heterologous expression. Application performance (D3) primarily refers to the substantial decline in performance of high-efficiency phosphate-solubilizing microbial strains screened under laboratory conditions when deployed in field environments, featuring two major performance drop-offs: the transition from axenic laboratory culture to greenhouse pot trials, and the scaling from controlled greenhouse conditions to open-field applications. Environmental safety (D4) covers horizontal gene transfer (HGT) risks, colonization and persistence of engineered strains, ecological perturbation and dispersal, and evolutionary escape.

The 4D-RFF constitutes a closed-loop architecture comprising twelve bidirectional feedback loops (**Figure 1**): genetic targets define the feasible space for engineering strategies (D1D2, F1), and outcomes from engineering implementation refine our understanding of targets (D2D1, F2); engineering strategies establish the baseline profile of field performance (D2D3, F3), while field failure informs iterative strategy revision (D3D2, F4); field performance data expose safety hazards (D3D4, F5), and safety design may compromise application efficacy (D4D3, F6); safety constraints restrict the choice of engineering strategies (D4D2, F7), and engineering strategies dictate the spectrum of safety risks (D2D4, F8); target selection predicts performance prospects (D1D3, F9), and performance data re-prioritize candidate targets (D3D1, F10); molecular features of targets determine the spectrum of safety risks (D1D4, F11), and safety constraints reshape target selection in reverse (D4D1, F12). This four-dimensional reciprocal feedback perspective constitutes the core original contribution that distinguishes this work from previous related reviews.

**Figure 1:**
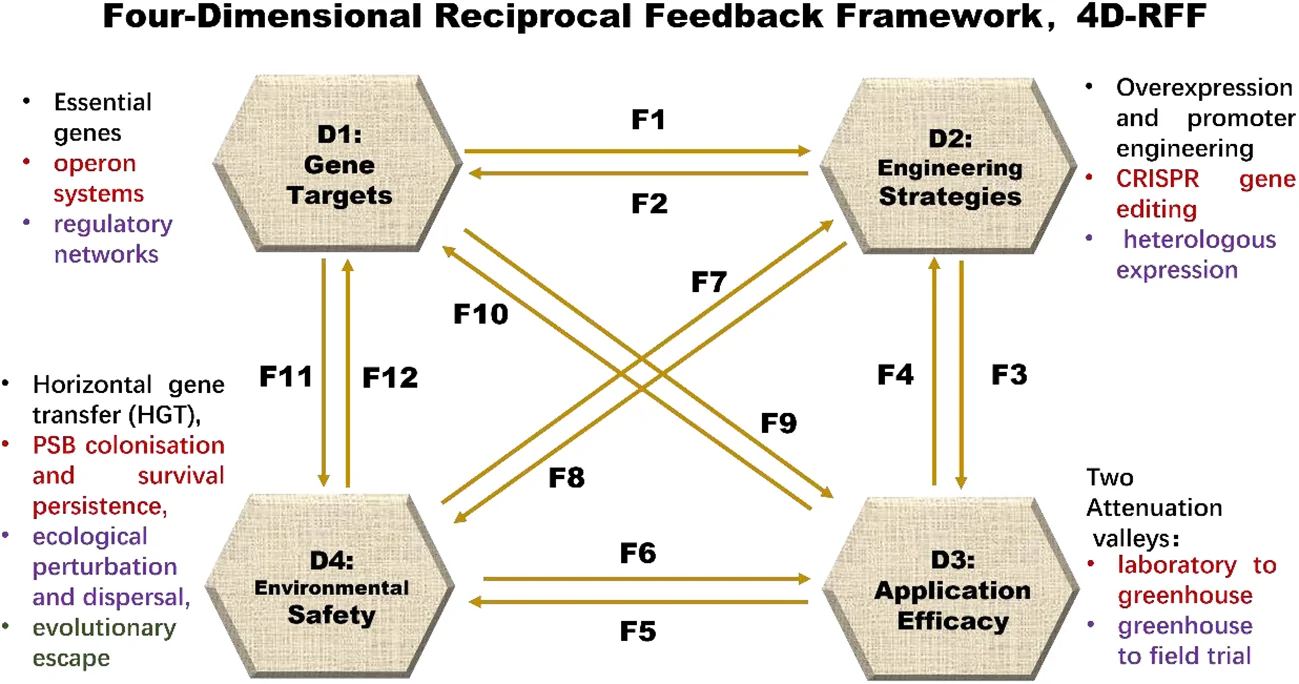
The four-dimensional reciprocal feedback framework (4D-RFF): twelve bidirectional reciprocal feedback relationships (F1–F12) among D1–D4. The four nodes in the figure represent genetic targets (D1), engineering strategies (D2), application performance (D3), and environmental safety (D4), respectively. Each pair of arrows denotes one reciprocal feedback relationship, with feedback loop identifiers labelled adjacent to the arrows.

### 1.4 Scope and structure of this Perspective

This article is framed as a Perspective, which synthesizes published evidence in an opinion-driven manner to propose and validate an original conceptual framework, rather than performing exhaustive retrieval and enumeration of the field literature. Literature searching and screening serve the construction and validation of the framework. The Four-Dimensional Reciprocal Feedback Framework functions both as an analytical tool for literature categorization and a means to identify knowledge gaps. In the **Supplementary Materials**, we present a four-dimensional coding analysis based on 163 cited references, which quantifies the frequency of different feedback relationships reported in the literature (**Supplementary Tables S1**-**S3**; **Supplementary Figure S1**), providing empirical evidence to support the reasoning of this framework.

This Perspective is organized as follows. First, we elaborate on the four dimensions (D1–D4) sequentially, with each section highlighting reciprocal feedback relationships with adjacent dimensions. We then introduce the unified decision-making framework of four-dimensional reciprocal feedback. Finally, we outlook future transitions from single-gene manipulation toward systems-level design, with dedicated discussion of evolutionary stability and quantitative prediction of escape frequency.

## 2 Dimension 1: genetic targets — molecular mechanisms and editable nodes

### 2.1 Inorganic phosphorus solubilization pathway: the *pqq* operon and *gcd* gene

Inorganic phosphorus solubilization constitutes one of the core functions of PSB, and its molecular basis mainly relies on the periplasmic oxidation pathway of glucose. After diffusing across the outer membrane into the periplasm, glucose is oxidized to gluconic acid by glucose dehydrogenase (Gcd). The resulting gluconic acid chelates metal cations such as calcium, iron and aluminum, thereby releasing immobilized phosphate (An and Moe, 2016). The rate-limiting step of this reaction lies not in the Gcd enzyme itself, but in its essential redox cofactor pyrroloquinoline quinone (PQQ) (Shen et al., 2012; Zhu and Klinman, 2020).

PQQ biosynthesis is encoded by the *pqq* operon, which typically comprises six genes from *pqqA* to *pqqF*, responsible for precursor peptide synthesis, cross-linking modification, oxidative cyclization, transport and final assembly, respectively (Shen et al., 2012). Studies have demonstrated that, within the genus *Pseudomonas*, the expression level of *pqq* genes is positively correlated with gluconic acid production and phosphate-solubilizing activity (An and Moe, 2016). Heterologous expression of the *pqq* gene cluster from *Acinetobacter calcoaceticus* in *Herbaspirillum seropedicae* Z67 endowed this strain with gluconate secretion capacity and phosphate-solubilizing function (Wagh et al., 2014). Co-expression of *gcd* and *pqqC* in *Pantoea agglomerans* has been validated as the molecular basis for the high-efficiency phosphate-solubilizing phenotype (Saadouli et al., 2021).

The gene composition and regulatory modes of the *pqq* operon vary across bacterial genera. In *Pseudomonas putida* KT2440, *pqq* genes are regulated by PqqR, whereas the PqqB protein may participate in oxidative modification of PQQ precursors (Choudhary et al., 2020). PQQ secretion also requires specific transporter systems; transporter genes involved in PQQ secretion have been identified in *Gluconobacter oxydans* (Wan et al., 2016). Environmental metagenomic data further reveal that *pqq* gene clusters are far more widely distributed among soil bacteria than in model strains, and the pqqC gene can serve as a molecular marker for detecting PSB (Enagbonma and Babalola, 2020; Joshi et al., 2023).

The *gcd* gene encodes a PQQ-dependent glucose dehydrogenase whose activity relies on PQQ supply. In *Pseudomonas*, *gcd* expression is controlled by the *PhoR*/*PhoB* two-component system (An and Moe, 2016). Gluconic acid can be further oxidized to 2-ketogluconic acid by gluconate dehydrogenase (GADH), which exhibits stronger metal-chelating capacity (Wang et al., 2022). In *Burkholderia* sp. Ha185, the *hemX* gene was proven critical for 2-ketogluconate production and the phosphate-solubilizing phenotype, indicating regulatory layers beyond the canonical *pqq–gcd* pathway (Hsu et al., 2015).

### 2.2 Organic phosphorus mineralization pathway: phosphatase and phytase gene families

PSB hydrolyze soil-borne organic-phosphorus compounds into inorganic phosphate via secreting diverse phosphatases. Bacterial phosphatases are categorized into three groups according to catalytic mechanisms and subcellular localization (Neal et al., 2017): Group A (PhoA-type) denotes periplasmic acid phosphatases; Group B represents periplasmic alkaline phosphatases; Group C refers to outer-membrane-anchored non-specific acid phosphatases. Three gene families, *phoA*, *phoD* and *phoX*, show divergent distribution patterns across marine and terrestrial ecosystems, among which *phoD* is most abundant in terrestrial soils (Huang et al., 2009; Ragot et al., 2015).

The alkaline phosphatase encoded by *phoD* is one of the most widespread enzymes mediating organic phosphorus mineralization in soils. Environmental metagenomic studies indicate that the abundance and diversity of *phoD* genes are tightly associated with soil organic phosphorus content, pH and vegetation type (Fraser et al., 2015). In karst ecosystems, vegetation restoration markedly enhances the diversity and community network connectivity of *phoD*-harboring microorganisms (Pan et al., 2024). Long-term fertilization can reshape the community composition of soil *phoD* genes, yet soil organic carbon, rather than phosphorus itself, acts as the primary driver structuring *phoD* communities (Hu et al., 2018). Land reclamation significantly increases topological parameters of *phoD* co-occurrence networks in calcareous soils (Zhang et al., 2023). In grassland afforestation systems, community assembly of *phoD* and *phoX* is mainly driven by soil moisture and pH (Chen et al., 2021). A direct correlation exists between *phoD* abundance and alkaline phosphatase activity, while *phoX* abundance is strongly affected by land-use type (Ragot et al., 2016).

Phytases constitute another key group of organic phosphorus-mineralizing enzymes, specialized in hydrolyzing phosphate ester bonds within phytic acid. Bacterial phytases fall into two major classes: *phyA* (histidine acid phosphatase family) and *phyC* (β-propeller phytase) (Hanif et al., 2015). Distinct phytases exhibit substantial differences in optimal pH and temperature ranges, which directly determine their catalytic efficiency in specific soil environments. Identification and functional validation of the β-propeller phytase (BPP) gene in *Bacillus subtilis* laid the molecular foundation for phytase engineering (Hanif et al., 2015). Furthermore, the β-propeller phytase encoded by the *bpp* gene and histidine acid phosphatase encoded by the *hap* gene are broadly distributed in soil bacteria and can be used as supplementary molecular markers to assess organic phosphorus mineralization function (Wan et al., 2020). **Table 1** systematically compares the molecular features and functional roles of different phosphatase gene families.

**Table 1:** Molecular features and functional roles of major phosphatase gene families in phosphate-solubilizing bacteria (Huang et al., 2009; Ragot et al., 2015; Neal et al., 2017).

Gene family	Subcellular localization	Substrate specificity	Regulatory mode	Representative genera
*phoA* (class A)	Periplasm	Broad-spectrum phosphomonoesters	PhoR/PhoB regulation	*E. coli*, *Pseudomonas*
*phoD* (class B)	Periplasm/extracellular	Alkaline phosphomonoesters	Phosphate-starvation inducible	*Bacillus*, *Pseudomonas*
*phoX* (class B)	Outer membrane	Alkaline phosphomonoesters	Phosphate-starvation inducible	*Rhizobium*, *Bradyrhizobium*
Class C NSAP	Outer-membrane anchored	Broad-spectrum organic phosphate	Constitutive/inducible	*Burkholderia*, *Enterobacter*
*phyA*/*phyC*	Extracellular secretion	Phytate-specific	Substrate inducible	*Bacillus*, *Aspergillus*

### 2.3 Regulatory networks: the *PhoR*/*PhoB* two-component system

Phosphorus metabolism in PSB is not constitutively expressed but coordinated by sophisticated regulatory networks. The *PhoR*/*PhoB* two-component regulatory system serves as the core regulatory hub for bacterial phosphorus starvation responses (Lüttmann et al., 2012). Under phosphorus-replete conditions, PhoR acts as a phosphatase to maintain PhoB in its dephosphorylated state. When environmental phosphorus concentration declines, PhoR switches to kinase activity and phosphorylates PhoB. Phosphorylated PhoB functions as a transcription factor to activate downstream phosphorus starvation-response genes, including the phosphorus transport system (*pstSCAB*), phosphatase genes and enzymes involved in phosphorus acquisition (Marzan and Shimizu, 2011). The regulatory network of the Pho regulon has been thoroughly characterized in *E. coli*, where the phosphate-specific transport system (Pst) and PhoU protein play critical roles in signal sensing (Caria et al., 2010).

Regulation via the *PhoR*/*PhoB* system exhibits strong environmental responsiveness. In *Escherichia coli*, the EIIA^Ntr^ protein of the PTS nitrogen regulatory system (PTS^Ntr^) binds PhoR and enhances its kinase activity, thereby coupling carbon and nitrogen metabolic status to the phosphorus starvation response (Lüttmann et al., 2012). In *Xanthomonas oryzae* pv. *oryzae*, constitutive activation of the PhoBR system conversely reduces strain fitness and virulence in phosphorus-rich host environments, indicating an adaptive cost associated with PhoBR-mediated regulation (Zheng et al., 2018). In *Streptomyces coelicolor*, phosphorylation kinetics of PhoP (the PhoB homolog) are modulated by multiple signal inputs, yielding a regulatory network far more complex than those in model strains (Santos-Beneit et al., 2016).

Furthermore, the *PhoR*/*PhoB* system is not the sole regulatory layer governing bacterial phosphorus metabolism. In *Amycolatopsis mediterranei*, GlnR, a global regulator of nitrogen metabolism, directly activates transcription of the phosphate-specific transporter operon *pstSCAB*, revealing cross-regulation between nitrogen and phosphorus metabolism (Zhang et al., 2018). In *Bacillus subtilis*, the PhoPR two-component system not only controls phosphorus metabolism but also coordinates cell-wall metabolism and antibiotic production (Botero et al., 2018). This finding suggests that engineering strategies for PSB may suffer limited efficacy if only phosphorus starvation pathways are targeted while cross-talk with carbon and nitrogen metabolism is ignored. Additionally, the roles of small regulatory RNAs in phosphorus starvation responses are being progressively uncovered (Stosiek et al., 2019).

### 2.4 Logic and limitations of target selection

In current genetic engineering research of PSB, target selection follows the principle of *known functionality*: genes that have been well characterized in model organisms are prioritized as engineering targets. Although reasonable at the initial research stage, this principle imposes three major limitations.

First, “model-organism bias” in target selection. Genes such as *pqq*, *gcd* and *phoD* have been most intensively studied in model genera including *Pseudomonas* and *Escherichia*. However, these model strains are not necessarily optimal rhizosphere colonizers for target crops (Kaur et al., 2021). Genomic analysis of the non-model strain *Pseudomonas asiatica* JP233 uncovered a unique phosphate-solubilizing mechanism centered on 2-ketogluconate as the core metabolic product (Wang et al., 2022). Genomic investigation of *Pseudomonas thivervalensis* SC5 identified multiple plant-growth-promoting genes (Nascimento et al., 2021). Second, the limitation of the “single-target mindset”. Most existing studies focus on overexpression or knockout of individual genes, yet phosphate solubilization represents a systemic process requiring coordination of multiple genes. PQQ biosynthesis requires the intact *pqqA*–*pqqF* operon, and gluconate production relies on synergy between Gcd and PQQ; insufficiency at any single step can become rate-limiting (Elena et al., 2014). Third, “disconnection between target selection and subsequent engineering strategies”. Molecular properties of targets — including operon architecture, genetic cooperativity, regulatory complexity and essentiality — constitute primary constraints for selecting engineering strategies, yet such constraints are frequently overlooked in current research.

In recent years, comparative genomics has opened new avenues for target discovery. Genomic analysis of *Pseudomonas asiatica* JP233 has clarified the genetic basis underlying its high phosphate-solubilizing capacity (Wang et al., 2022). Genomic profiling of *Pseudomonas thivervalensis* SC5 has revealed its diverse plant-growth-promoting genes (Nascimento et al., 2021). Genome-scale metabolic models (GEMs) provide powerful tools to systematically dissect PSB metabolic networks; reconstruction of the genome-scale metabolic network in *Pseudomonas fluorescens* has uncovered regulatory circuits of central carbon metabolism (Huang and Lin, 2019). Nevertheless, the real challenge lies in identifying “high-payoff targets” from numerous candidate genes — namely, genetic nodes that exert the largest impacts on phosphate-solubilizing function while remaining tractable for engineering. Addressing this challenge requires systematic integration of multi-omics datasets and functional validation.

It should be further clarified that this Perspective does not merely enumerate genes; instead, it proposes screening criteria for “high-reward targets”. A candidate gene is prioritized only if it simultaneously satisfies the following four requirements: (i) clear functional gain — it makes quantifiable contributions to phosphate-solubilizing flux or field growth-promoting performance; (ii) high engineering feasibility — it has well-defined operon architecture and accessible transformation and editing tools; (iii) compatibility with downstream strategies — single genes are suitable for overexpression or promoter replacement, whereas multi-gene pathways are amenable to genomic integration or heterologous module deployment; (iv) manageable safety risks — it does not involve plasmid transfer elements or high-risk resistance markers. For instance, the *pqq* operon and *gcd* are repeatedly selected as targets precisely because they directly govern the flux of gluconate, a core phosphate-solubilizing metabolite (D1). This property further constrains feasible engineering strategies (D2), predicts the upper limit of inorganic-phosphate solubilization under field conditions (D3), and confers relatively low HGT risks owing to their status as endogenous functional modules (D4). By comparison, *phoD* is better suited for soils dominated by organic phosphorus, and its priority should be dynamically adjusted according to the phosphorus-speciation profile of target soils. These criteria create a closed loop between target-gene selection and dimensions D2-D4, rather than yielding an isolated list of molecular candidates.

## 3 Dimension 2: engineering strategies — performance boundaries of three technical routes

### 3.1 Overexpression and promoter engineering

Overexpression of endogenous genes represents the most widely adopted strategy for PSB engineering. In *Herbaspirillum seropedicae* Z67, introduction of additional copies of the *pqq* gene cluster via plasmids increased gluconate secretion to 23.5 mM and markedly elevated phosphate-solubilizing activity (Wagh et al., 2014). In *Pseudomonas fluorescens*, heterologous expression of phosphoenolpyruvate carboxylase (PEPC) redirected glucose metabolic flux and improved both biomass accumulation and phosphate-solubilizing capacity (Buch et al., 2009). Wagh et al. further co-expressed *pqq* and gluconate dehydrogenase (*gad*) genes in *H. seropedicae* Z67, raising phosphate-solubilizing activity to 457.7 μM (Wagh et al., 2016). In *Enterobacter asburiae* PSI3, overexpression of the *gad* operon from *Pseudomonas putida* KT2440 endowed the strain with the capacity to produce 2-ketogluconate and dissolve phosphate rock (Ribeiro et al., 2020).

Promoter engineering constitutes an advanced variant of overexpression strategies. Replacing native promoters with stronger constitutive or inducible promoters enables tunable control over the magnitude and timing of target gene expression (Jin et al., 2019; Xu et al., 2019). Construction of promoter libraries and RBS optimization are widely applied in metabolic engineering within synthetic biology (Zhang et al., 2019; Li et al., 2021a). Nevertheless, the application of promoter engineering in PSB remains in its infancy. Most PSB are non-model strains with poorly characterized promoter libraries. Studies in *Streptomyces lividans* have demonstrated that bacterial promoters from distinct sources can differ in activity by several orders of magnitude when expressed in heterologous hosts (Zhao et al., 2018), indicating that promoter selection requires host-specific systematic optimization. Inducible expression systems — such as promoters controlled by arabinose, IPTG or quorum-sensing signals — have been deployed in metabolic engineering to decouple microbial growth from product formation (Yang et al., 2018; Feng and Marchisio, 2021).

Overexpression strategies are constrained by three performance boundaries. First, metabolic burden: maintenance of high-copy plasmids and synthesis of overexpressed proteins consume substantial ATP and amino acids, potentially reducing strain growth rate and rhizosphere competitiveness (Tan et al., 2020). Second, plasmid stability: plasmid loss can occur at high rates under field conditions in the absence of antibiotic selection pressure. Andreeßen et al. designed a carbon-source-dependent plasmid addiction system in *Shimwellia blattae* to mitigate plasmid loss (Andreessen et al., 2014), yet the applicability of this strategy in soil environments remains to be validated. Toxin–antitoxin (TA) systems have been extensively investigated as plasmid maintenance modules (Mijakovic et al., 2005). Third, regulatory imbalance: overexpression may disrupt native cellular metabolic homeostasis, triggering stress responses and imposing adaptive costs (Fernandez-Bunster and Pavez, 2022).

### 3.2 CRISPR-Cas gene editing

The CRISPR-Cas system delivers unprecedented precision for genetic engineering of PSB. Cas12a (Cpf1) is one of the most widely used Cas effector proteins for bacterial genome editing in recent years. Its preference for T-rich PAM sequences affords broader targeting scope in bacterial genomes with low GC content (Yan et al., 2017; Meliawati et al., 2021). Compared with Cas9, Cas12a generally exhibits lower toxicity in non-model hosts and can self-process crRNA arrays, facilitating multiplexed editing (Paul and Montoya, 2020; Chua and Collins, 2022). Efficient genome editing mediated by Cas12a has been achieved in *Escherichia coli (*Ao et al., 2018), *Thioalkalivibrio versutus (*Hao et al., 2023), *Stenotrophomonas (*Meng et al., 2023), and *Cyanothece* PCC 7425 (Khan et al., 2026). The discovery and engineering of novel Cas12a nucleases such as AiEvo2 have expanded the PAM-recognition scope and editing efficiency (Sharrar et al., 2024). Furthermore, the RecET-assisted system enables efficient manipulation of large-fragment DNA in *Zymomonas mobilis*, offering a new toolbox for the integration of multi-gene pathways (Zhang et al., 2025).

Nevertheless, CRISPR editing faces unique challenges when applied to PSB. First, most PSB are non-model organisms with incomplete genomic information and lack robust transformation protocols. Elmore et al. developed a high-throughput method based on iterative site-specific genomic integration, offering new insights for engineering non-model strains (Elmore et al., 2023). Second, heterologous expression of Cas proteins can exert intrinsic toxicity to host cells. FnCas12a-based systems show reduced toxicity relative to SpCas9 in *Streptomyces*, making them the preferred option for editing non-model strains (Meliawati et al., 2021). Third, off-target effects of CRISPR editing are difficult to assess without complete genomic data and may introduce unintended genomic variations. In *Magnaporthe oryzae*, Cas12a-induced DNA double-strand breaks generate diverse mutation spectra via multiple repair pathways (Huang et al., 2022). In addition, the SEVA-Cpf1 platform provides standardized CRISPR-Cas12a vectors for cyanobacteria, yet its editing efficiency is limited by the polyploid nature of host strains; multiple rounds of screening are usually required to obtain homozygous edited genomes (Baldanta et al., 2022). This experience offers valuable lessons for editing polyploid PSB.

### 3.3 Heterologous expression

Heterologous expression introduces exogenous phosphorus-solubilizing genes into a target host strain to endow or enhance its phosphate-solubilizing function. A representative study by Shulse et al. screened and recoded 82 phylogenetically diverse phytase genes, then integrated them into the genomes of three rhizosphere-colonizing bacteria. They obtained 41 engineered strains with high phytate-hydrolyzing capacity, among which 12 strains significantly promoted the growth of *Arabidopsis thaliana* when phytate served as the sole phosphorus source (Shulse et al., 2019). This work demonstrates the great potential of synthetic biology for systematically constructing novel phosphate-solubilizing strains.

Heterologous expression encounters three major challenges. First, codon bias: codon usage frequencies differ substantially across bacterial genera (Elena et al., 2014). Second, protein folding and function: heterologous proteins may fail to fold correctly or lack essential post-translational modifications in the recipient host. Third, metabolic compatibility: introduction of exogenous functional modules may perturb the native metabolic network of the host. Within synthetic biology, modular design and construction of orthogonal genetic systems provide strategies to address these hurdles (Rawat et al., 2021; Silva Vale et al., 2022).

### 3.4 Condition matrix and decision logic for the three routes

A systematic comparison of the applicable conditions and performance boundaries across the three engineering-strategy pathways reveals an important structural feature (**Table 2**): the dominant strategy currently used for engineering phosphate-solubilizing microorganisms — overexpression/promoter engineering — ranks lowest precisely in the two most critical attributes for field-relevant performance, namely genetic stability and field applicability. CRISPR-mediated editing exhibits distinct advantages in genetic stability and field suitability, yet its application in phosphate-solubilizing microorganisms remains in its early stages. The heterologous-expression strategy offers unique strengths in functional modular design, but it faces dual challenges of codon compatibility and metabolic compatibility. It should be noted that **Table 2** presents a qualitative comparison built upon published findings, rather than a weighted quantitative scoring system. The meanings of each column dimension (applicable targets, host requirements, genetic stability, metabolic burden, throughput, and field applicability) are defined by the text within the table. No artificial weighting factors or composite rankings are assigned in this manuscript; descriptors such as “low” and “high” represent only relative qualitative judgments.

**Table 2:** Applicable conditions and performance boundaries of three genetic-engineering strategies.

Evaluation dimension	Overexpression/promoter engineering	CRISPR gene editing	Heterologous expression
Applicable targets	Single genes, endogenous genes	Any gene, regulatory elements	Multi-gene pathways, functional modules
Host requirements	Operable promoter system required	Transformation method and genome information required	Codon compatibility required
Genetic stability	Low (plasmid loss-prone)	High (genomic integration)	Medium (depends on integration mode)
Metabolic burden	Medium to high	Low (fixed after editing)	Medium to high
Editing throughput	Medium	Low (locus-by-locus editing)	High (modular design)
Field applicability	Low (selection pressure required)	High (edited strains stably inherited)	Medium (environmental adaptation requires validation)
Representative studies	(Wagh et al., 2014; Wagh et al., 2016)	(Meliawati et al., 2021)	(Shulse et al., 2019)

### 3.5 A three-tier decision logic for engineering strategy selection

Building on the above analysis, we propose a three-tier decision framework for engineering strategy selection. The first tier concerns gene essentiality: essential genes cannot be knocked out and must be regulated via overexpression or promoter replacement, whereas non-essential genes can be modified through CRISPR knockout or functional substitution. The second tier concerns genomic organization of targets: single-gene targets are amenable to all three strategies; multi-gene operons are best suited for overexpression or heterologous expression; and dispersed multi-gene pathways favor genomic integration or heterologous expression modules. The third tier concerns application-stage constraints: plasmid-based expression instability is acceptable during laboratory validation; genomic integration is recommended at the greenhouse trial stage; and genetic stability plus biosafety requirements become mandatory at the field release stage. The key implication of this decision logic is that engineering strategy selection is not a matter of “choosing the best technology”, but rather an optimization problem of “identifying the optimal combination under given constraints”.

## 4 Dimension 3: application performance — translation bottlenecks from laboratory to field

### 4.1 Performance gaps between greenhouse and field trials

High-efficiency PSB strains screened under laboratory conditions often experience substantial performance attenuation when deployed in field environments. Under controlled conditions, PSB can achieve phosphate-solubilizing efficiencies at the millimolar level (Yadav et al., 2013). In greenhouse pot experiments, PSB inoculation typically increases crop biomass by 15%–40% (Anzuay et al., 2017; Timofeeva et al., 2022). Yet when the same strain proceeds to field trials, efficacy often drops markedly, or even loses statistical significance. The association between inoculant performance and the structure of native soil microbial communities is a key variable for predicting inoculant efficacy (Imam et al., 2021).

Meyer et al. reported that *Pseudomonas protegens* CHA0 failed to significantly enhance wheat uptake of ^33^P-labeled phosphorus from synthetic hydroxyapatite in calcareous soil (Meyer et al., 2019). Israeli et al. showed that microbial inoculants were far less effective than grassland diversity management strategies in intensive grassland systems (Ikoyi et al., 2024). In sunflower cultivation, *Enterobacter* sp. Fs-11 exhibited good colonization and plant-growth-promoting traits in sterile sand culture, but its efficacy declined significantly in natural soil (Shahid et al., 2012). These studies reveal a core issue: the discrepancy between laboratory conditions and field environments is not quantitative but qualitative — precisely the factors excluded from laboratory settings are those that determine field performance, including microbial competition, abiotic stress fluctuations, soil heterogeneity and dynamic plant responses (Rosa et al., 2020; Blanco-Romero et al., 2023).

### 4.2 Rhizosphere colonization: the first translation bottleneck

Rhizosphere colonization is a prerequisite for engineered PSB to exert their functions. It is a multi-step process encompassing chemotactic migration to the root surface, root attachment, biofilm formation and sustained rhizosphere persistence (Romano et al., 2020). Introduced engineered strains face intense competition from indigenous microbial communities. Studies have shown that survival rates of introduced strains in soil typically decline sharply within days after inoculation and may fall below detection limits after several weeks (Ambrosini et al., 2015). Root exudates play a critical role in shaping the rhizosphere microbiome; different plant species and genotypes recruit specific microbial communities through distinct root exudate profiles (Chaparro et al., 2012; Liu et al., 2019).

The factors influencing rhizosphere colonization capacity are extraordinarily complex. First, strain–crop specificity: colonization performance of a single strain varies markedly across crop rhizospheres. Ferreira et al. performed meta-omic analysis of *Azospirillum brasilense* inoculants and demonstrated that the diversity of native soil microbial communities significantly affects the establishment of introduced inoculants (Ferrarezi et al., 2023). Second, soil physicochemical properties: pH, organic matter content, moisture status and nutrient levels all modulate colonization efficiency (Romano et al., 2020). Tienda et al. developed *Pseudomonas*-based rhizosphere-colonizing biocontrol consortia under high-temperature stress, demonstrating a viable pathway to improve colonization efficiency by screening and combining strains with complementary colonization strategies (Tienda et al., 2024). Bacterial chemotaxis and motility — including flagellum-mediated swimming and type IV pilus-mediated twitching — represent key functional traits for rhizosphere colonization (Scharf et al., 2016).

### 4.3 Sustained functional expression: the second translation bottleneck

Even when engineered strains successfully colonize the rhizosphere, the persistence of phosphate-solubilizing function under field conditions remains a formidable challenge. The foremost issue is plasmid loss: under field conditions lacking antibiotic selection pressure, segregational plasmid instability can abolish function within a few generations (Andreessen et al., 2014). Second, expression regulation of functional genes: the *PhoR/PhoB* system activates downstream gene expression under phosphorus starvation, but phosphorus distribution in field soils is highly heterogeneous; phosphorus concentrations in rhizosphere microzones may far exceed those in bulk soil, potentially causing engineered strains to downregulate phosphate-solubilizing genes in “phosphorus-rich” rhizosphere microsites (Marzan and Shimizu, 2011). Third, long-term effects of metabolic burden: constitutive high-level expression of exogenous functional proteins consumes substantial cellular resources, which may reduce the ecological fitness of engineered strains over time (Tan et al., 2020; Fernandez-Bunster and Pavez, 2022). Within synthetic biology, quantification and management of metabolic burden have emerged as central considerations in engineered strain design (Snoeck et al., 2024).

**Figure 2** visualizes these two translation bottlenecks and the feedback loop of ALE pre-adaptation.

**Figure 2:**
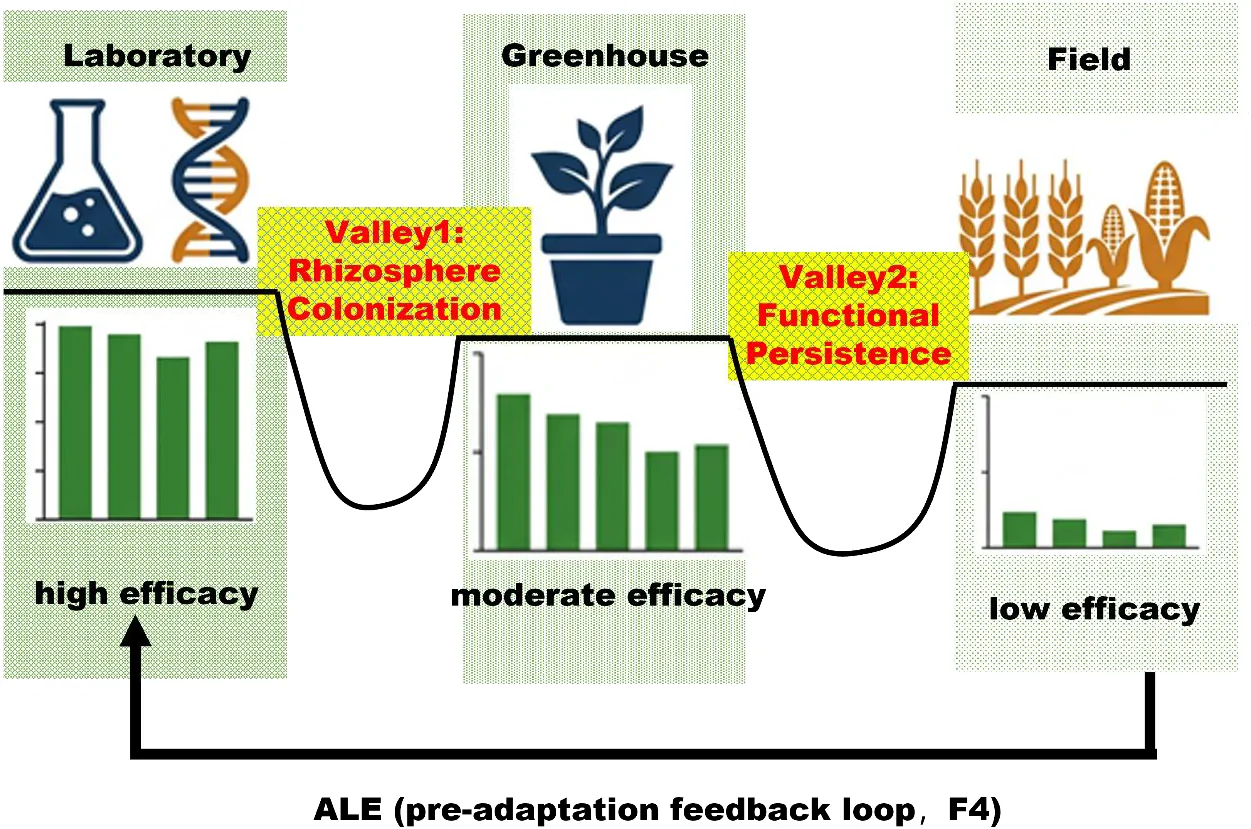
Two translation bottlenecks and the F4 feedback loop along the laboratory-to-field pipeline. The figure marks the translation bottlenecks responsible for progressive performance decay across three stages: laboratory, greenhouse and field. It also illustrates soil microcosm-based ALE pre-adaptation as the practical implementation of the F4 feedback loop (performance-to-strategy).

### 4.4 *In situ* soil evolution and adaptive laboratory evolution: the critical feedback for strategy revision (F4)

A dimension severely overlooked in existing research is the influence of “*in situ* soil evolution” on the field performance of engineered strains. The selective pressures encountered by engineered strains in soil environments differ drastically from laboratory conditions: low nutrient availability, oxidative stress, fluctuating osmotic pressure and microbial competition collectively impose strong selection, which may drive mutational inactivation or downregulation of engineered genes. Even genomically integrated strains constructed via CRISPR editing may acquire genotypes with attenuated function during long-term field persistence through IS-element transposition, homologous recombination or point mutation. This phenomenon is fundamentally an issue of evolutionary stability: there exists a fundamental tension between the engineered “designed function” and the strain’s evolutionary fitness. Evolutionary dynamics of microbial genomes under environmental selection have been widely studied; mutational hotspots and IS-element density within genomes serve as key parameters for predicting evolutionary trajectories (Yang et al., 2018; Deryabin et al., 2019).

Nevertheless, this challenge also unveils opportunities. Adaptive laboratory evolution (ALE) has been broadly adopted in microbial metabolic engineering to screen mutant strains with superior traits (Dragosits and Mattanovich, 2013; Lapanse et al., 2022), yet its application in PSB engineering remains nearly unexplored. ALE enriches adaptive mutants via serial passaging under defined selective pressure, and has achieved remarkable success in improving thermotolerance, substrate utilization spectra and stress resistance (Lapanse et al., 2022). Integration of ALE with metabolic engineering and synthetic biology has become an important paradigm for industrial microbial breeding (Zhang et al., 2019).

It is important to candidly acknowledge, however, a key theoretical limitation of ALE pre-adaptation: adaptive mutants isolated under liquid laboratory culture do not necessarily retain competitive advantages in complex soil environments. Traits selected under laboratory adaptation and soil adaptation may conflict, or even exhibit antagonistic pleiotropy — genotypes optimized for fitness in chemostats may perform poorly in soil due to nutrient limitation, spatial heterogeneity and interspecific competition. Work by Chen and Zhang demonstrated that molecular signatures of adaptation observed under constant laboratory conditions are often masked by antagonistic pleiotropy in fluctuating environments (Chen and Zhang, 2020). Local-adaptation experiments on soil bacteria by Belotte et al. revealed strong genotype-by-environment interactions between growth rates in laboratory culture and soil extracts (Belotte et al., 2003). Studies by Goddard and Bradford on adaptive responses of natural microbial populations under carbon and nitrogen limitation also indicate that traits selected through short-term laboratory adaptation do not necessarily reflect fitness in natural settings (Goddard and Bradford, 2003). Renda et al. found that populations of *Acinetobacter baylyi* ADP1 evolved in LB liquid medium displayed markedly different fitness in media supplemented with forest soil metagenomic DNA, and genomic instability during laboratory evolution could lead to loss of key traits (Renda et al., 2014). Collectively, these studies reveal a core insight: soil is an ecosystem characterized by high spatial heterogeneity, fluctuating nutrient availability and intense interspecific competition, fundamentally distinct from liquid chemostat cultures.

We therefore propose that ALE pre-adaptation should not be performed in conventional liquid chemostat systems, but via serial passaging in microcosms containing target soil, root exudates and indigenous microbial communities. Although this “soil-microcosm ALE” strategy has lower throughput, it is more likely to recover mutants with genuine adaptive advantages under field conditions. Work by Van Dyke and Prosser demonstrated that pre-incubation in sterile soil carriers significantly improved survival of *Pseudomonas fluorescens* in soil (Dyke and Prosser, 2000). Megharaj et al. showed that soil-adapted strains exhibited substantially higher survival in non-sterile soil than strains adapted in liquid media (MegharaJ et al., 1997). Long-term ALE pre-adaptation of engineered strains in microcosms mimicking target soil conditions uses natural selection to enrich adaptive mutants that both maintain phosphate-solubilizing function and tolerate field-associated stresses. The core logic of this strategy is: rather than “over-engineering” a strain unprepared for field environments in the laboratory, harness the screening power of evolution for pre-adaptation prior to environmental release. Microcosm experiments have proven effective at recapitulating key field selective pressures while offering good experimental reproducibility (Blanco-Romero et al., 2023). Future work may further adopt *in situ* evolution technologies such as adaptation chips (aChips) to construct physical models of soil microenvironments within microfluidic devices, enabling high-throughput screening for spatial heterogeneity.

This strategy simultaneously implements the critical F4 feedback loop: field performance data inform strategy revision. When field trials reveal performance decay of engineered strains, decaying isolates can be recovered, sequenced and analyzed to identify mutational hotspots responsible for loss of function. These findings then guide revisions to CRISPR editing strategies — for example, introducing synonymous mutations at mutational hotspots to eliminate IS-element insertion sites, or adding copy redundancy for key functional genes. Varivarn et al. showed that *Pseudomonas fluorescens* Pf0–1 activates a set of soil-specific genes not expressed in laboratory media; these genes are critical for understanding field fitness (Varivarn et al., 2013). ALE pre-adaptation and field decay analysis together form an iterative “design–test–evolve–revise” closed loop.

### 4.5 Crop specificity and application examples

The application performance of engineered PSB exhibits strong crop specificity and environmental dependence. Efficacy of the same strain can vary several-fold across different crops. Mechanisms underlying this variation include differences in root exudate composition, rhizosphere pH and plant–strain signal crosstalk (Chaparro et al., 2012; Anzuay et al., 2017). Comparative studies on peanut and maize show that PSB exert stronger growth-promoting effects on peanut than on maize (Anzuay et al., 2015).

**Table 3** systematically summarizes application examples of engineered PSB and high-performance native PSB across different crops. A striking pattern emerges: PSB generally deliver greater growth benefits in leguminous crops than in gramineous crops, with intermediate performance in solanaceous crops. This difference is tightly linked to root exudate profiles: legumes secrete more organic acids and flavonoids, generating a more favorable rhizosphere microhabitat for PSB colonization (Chaparro et al., 2012). Furthermore, field validation data for major global grain crops such as maize and rice are far less abundant than for peanut and soybean, representing a critical research gap to be addressed.

**Table 3:** Application cases of engineered and native high-efficiency phosphate-solubilizing bacteria across crop species.

Crop type	Representative strains	P-solubilization mechanism	Maximum growth-promoting effect	Experimental conditions
Peanut	*Enterobacter* sp. J49, *Serratia* sp. S119	Organic acids + phosphatase	Biomass +37%, yield +25%	Field (Anzuay et al., 2015; Anzuay et al., 2017)
Soybean	*Pseudomonas* spp., *Bacillus* spp.	Organic acids + siderophores	Biomass +15-28%, P uptake +20%	Greenhouse/field (Bakhshandeh et al., 2014)
Maize	*Azospirillum brasilense*, *Pseudomonas* spp.	Organic acids + phytohormones	Yield +8-15%, 20% reduction in P fertilizer input	Field (Ferrarezi et al., 2023; Vega-Camarillo et al., 2023)
Wheat	*P. protegens* CHA0, *Bacillus* spp.	Organic acids + phosphatase	Biomass +5-12%, P uptake not significant	Field (Meyer et al., 2019; Rizvi et al., 2021)
Rice	*Burkholderia* spp., *Enterobacter* spp.	Organic acids + phytase	Biomass +10-20%, yield +8-12%	Greenhouse/field (Zhao et al., 2013; You et al., 2019)
Tomato	*Pseudomonas* spp., *Bacillus* spp.	Organic acids + siderophores + phytohormones	Biomass +18-30%, fruit yield +15%	Greenhouse (Santoyo et al., 2021)
Potato	*Pseudomonas* spp., *Burkholderia* spp.	Organic acids + phosphatase	Tuber yield +12-22%, P content +15%	Greenhouse/field (Saeed et al., 2021)
Sunflower	*Enterobacter* sp. Fs-11, *Pseudomonas* spp.	Organic acids + ACC deaminase	Biomass +20-30% (sand culture), attenuated under field conditions	Sand culture/greenhouse (Shahid et al., 2012)

Environmental dependence also acts as a key factor limiting application performance. In acidic soils, iron- and aluminum-bound phosphorus is more readily solubilized, yet the growth of PSB itself may be inhibited by low pH (Jorquera et al., 2010). In calcareous soils, solubilization of calcium-bound phosphorus requires higher concentrations of organic acids, and free calcium ions may react with secreted organic acids (Meyer et al., 2019). Soil pH not only affects PSB activity but also indirectly modulates organic phosphorus mineralization efficiency by regulating *phoD* gene expression and alkaline phosphatase activity (Ragot et al., 2016). Phosphorus speciation distribution differs markedly across soil types: calcium-bound phosphorus dominates in calcareous soils, whereas iron- and aluminum-bound phosphorus are more abundant in acidic soils (Ducousso-Détrez et al., 2022).

The systematic comparison of engineered PSB (**Table 4**) performance between greenhouse and field conditions clearly demonstrates stepwise declines in phosphorus solubilization capacity, crop biomass increment and data reproducibility along the pipeline from laboratory to field. This multi-dimensional evaluation framework not only reflects the comprehensive contributions of engineered PSB more accurately, but also delivers refined feedback for the optimization of engineering strategies.

**Table 4:** Performance comparison of engineered phosphate-solubilizing bacteria under laboratory, greenhouse-pot and field conditions.

Evaluation metric	Laboratory	Greenhouse pot	Field trial
P solubilization (µM)	200-500	50-200	10-50
Increase in crop biomass (%)	N/A	15-40	0-15
Duration of functional activity	Days	Weeks	Weeks to months
Root colonization success	N/A	High	Low to medium
Data reproducibility	High	Medium	Low
Representative studies	(Yadav et al., 2013)	(Anzuay et al., 2017)	(Meyer et al., 2019; Ikoyi et al., 2024)

N/A, not applicable/not assessed. The unit of P solubilization (originally given as M) has been converted to µM in line with standard journal usage.

### 4.6 Innovation in evaluation paradigms for application performance

Current assessment of PSB application performance centers on “yield increment”, and this paradigm carries fundamental limitations. We propose that performance evaluation should shift from a yield-centric paradigm to a multi-dimensional performance framework consisting of three tiers: functional performance (phosphorus solubilization amount, phosphorus uptake, phosphorus use efficiency), ecological performance (rhizosphere microbial community structure, functional gene expression profiles, changes in soil enzyme activities), and systemic performance (crop yield, quality, stress tolerance, and the potential for reduced phosphorus fertilizer application).

## 5 Dimension 4: environmental safety — a neglected design parameter

The term “environmental safety” (D4) discussed in this Perspective encompasses four sub-dimensions: (1) horizontal gene transfer (HGT) risk — the spread of engineered genetic elements into indigenous microbial communities via conjugation, transformation, and transduction; (2) colonization and persistence — the survival, root-rhizosphere colonization, and long-term residence of engineered strains in target soils; (3) ecological perturbation and dispersal — impacts of engineered strains on indigenous community structure, functional redundancy, and soil food webs, as well as their spatial dispersal; (4) evolutionary escape — the probability that biocontainment circuits fail and strains undergo “de-domestication” to regain environmental fitness.

### 5.1 Risks of horizontal gene transfer

Following field release of genetically engineered PSB, engineered genetic elements they carry may spread into indigenous microbial communities via horizontal gene transfer (HGT). HGT constitutes one of the core driving forces of bacterial evolution and proceeds in soil through three mechanisms: conjugation, transformation and transduction (Babalola, 2014). Soil is regarded as a “hotspot” environment for HGT; its complex microbial community structure and abundant mobile genetic elements provide natural conditions for horizontal gene exchange (Selbitschka et al., 1992; Nielsen et al., 1997).

Quantification of three parameters is critical for HGT risk assessment: transfer frequency, post-transfer selective advantage, and dispersal range. Under ideal laboratory conditions, conjugation frequencies can reach 10^-^¹ to 10^-^³ per recipient cell, yet *in situ* frequencies in soil are typically several orders of magnitude lower (Wright et al., 2013). The post-transfer selective advantage depends on whether the engineered gene confers fitness benefits to recipient strains. If the engineered gene also enhances phosphorus acquisition in indigenous bacteria, these recipients may gain competitive advantages under phosphorus-limited conditions. Antibiotic resistance marker genes widely used in PSB engineering represent one major source of HGT risk. Alternatives include auxotrophic complementation markers, toxin–antitoxin system markers and fluorescent reporter gene markers (Whitford et al., 2018). Biosafety assessment of transgenic microorganisms has become a central topic within international regulatory frameworks (Singh et al., 2006; Committee et al., 2020).

### 5.2 Ecological perturbation: short-term reversible effects and long-term cumulative risks

Upon release into soil, engineered PSB may perturb the structure and function of indigenous microbial communities through multiple pathways. Short-term disturbances, such as transient declines in indigenous microbial biomass after inoculation, are generally considered reversible. However, empirical evidence for long-term cumulative effects remains extremely limited. A review by Ambrosini and Passaglia noted that inoculation with free-living active bacteria usually induces only short-lived impacts on soil microbial communities, whereas rhizobial inoculants or inoculation under stress conditions may produce long-term effects (Ambrosini et al., 2015).

A critical knowledge gap is that most studies examining ecological effects of engineered PSB last no longer than a single growing season, lacking multi-year long-term monitoring data. More importantly, ecological perturbation is not limited to shifts in community composition. From a functional ecology perspective, introduction of engineered PSB may trigger cascading effects via five pathways:

1. Trophic cascading: elevated phosphorus availability may alter carbon:phosphorus stoichiometry within rhizosphere microbial communities, drive turnover of dominant taxa, and further modify soil organic matter decomposition and nutrient cycling rates;
2. Erosion of functional redundancy: indigenous PSB communities may lose functional redundancy due to competitive exclusion by engineered strains, reducing community resilience to stress;
3. Reprogrammed plant–microbe interactions: shifts in rhizosphere phosphorus concentrations may modify root exudate profiles and reshape plant–microbe signaling networks (Chaparro et al., 2012);
4. Disturbance to soil food webs: changes in PSB populations may affect protozoan and nematode communities through predator–prey interactions (Rosa et al., 2020);
5. Coupled biogeochemical cycles: altered phosphorus cycling may modify carbon and nitrogen transformation rates via microbial metabolic networks.

These cascading effects have barely been systematically evaluated in existing literature, representing deeper ecological risks beyond simple shifts in community structure.

Pei et al. argued that synthetic biology safety standards must shift from technology-feasibility-driven frameworks toward risk-assessment-driven frameworks (Pei et al., 2022). In the bioeconomy era, advances must be pursued simultaneously along two dimensions: “safety-by-design” and technical feasibility (Arnolds et al., 2021). Notably, assessment of ecological perturbation should not focus solely on adverse effects; it is necessary to distinguish acceptable perturbations (e.g., short-term reversible community fluctuations) from unacceptable perturbations (e.g., irreversible species loss or functional degradation). Such distinction requires thorough understanding of baseline states and natural fluctuation ranges of soil ecosystems. Currently, the field lacks consensus criteria defining what constitutes an acceptable ecological disturbance, forming a major gap in regulatory science.

### 5.3 Quantitative models for evolutionary escape: from technology cataloging to quantitative design

Current discussions of biological containment strategies mostly remain at the level of technology enumeration—listing toxin–antitoxin systems, auxotrophy, environmentally triggered kill switches, etc.—without quantitative analysis of evolutionary escape. This constitutes a core barrier preventing biological containment from progressing from laboratory concepts to field deployment.

Evolutionary escape is fundamentally a population-genetics problem. For an engineered strain carrying a lethal genetic circuit, the inactivation mutation rate of the circuit and effective population size jointly determine the probability of escape mutant emergence. Under mutation–selection balance, the equilibrium frequency of escape mutants in the population is approximated as a function of the selection coefficient. For a single toxin–antitoxin system, the per-site inactivation mutation rate is on the order of specified magnitude per generation. If the lethal circuit is controlled by a single gene, escape mutants will almost certainly emerge within field-sized populations. Comparative experiments by Halvorsen et al. in *Pseudomonas fluorescens* demonstrated that even optimized nuclease-based toxin suicide switches yield escape frequencies within a defined range (Halvorsen et al., 2022), far above the sub-detection-limit standard required for field application. Bull and Barrick systematically analyzed evolutionary stability of engineered microbes and showed that decay rates of engineered functional modules depend on combined effects of mutation rate, selection coefficient and population size (Bull and Barrick, 2017). A recent critical review by George et al. on genetic biocontainment highlighted the lack of standardized escape-frequency measurement protocols; reported escape frequencies vary across laboratories by multiple orders of magnitude, and no existing containment strategy has undergone long-term field validation (George et al., 2024).

Reducing escape frequency to acceptable levels requires multi-layer redundant design, with each layer adopting orthogonal molecular mechanisms so that no single mutation can bypass multiple containment layers simultaneously. We propose the “Multiple Redundant Orthogonal Dependence (MROD)” design principle for essential genes:

1. Select three or more essential genes (e.g., *dnaE*, *gyrB*, *rpoB*) and design orthogonal transcriptional dependencies for each; expression of each essential gene is controlled by distinct non-natural inducers (e.g., non-canonical amino acids, synthetic small molecules, orthogonal ribosomes).
2. Deploy distinct molecular mechanisms for each regulatory circuit (e.g., allosteric transcription factors, riboswitches, intein-mediated protein splicing) to ensure no single mutational type can inactivate multiple containment layers at once.
3. Introduce spatial segregation of redundancy: distribute containment circuits across distinct genomic loci to reduce the probability that homologous recombination deletes multiple circuits simultaneously.

Mandell et al. computationally redesigned essential enzymes to render engineered strains dependent on non-canonical amino acids for survival, achieving escape frequencies below detection limits under laboratory conditions (Mandell et al., 2015). Orthogonal ribosome “biological firewalls” create genetic isolation by designing orthogonal ribosome–mRNA pairings such that translation of engineered genes relies exclusively on orthogonal ribosomes (Jia et al., 2017). Intein-mediated temperature-controlled homing endonuclease systems regulate DNA cleavage activity via temperature-responsive protein splicing (Foo et al., 2024). Stirling et al. developed rationally designed containment strategies based on orthogonal ribosomes to achieve robust genetic isolation of engineered strains (Stirling et al., 2017; Stirling et al., 2020). CRISPR-Cas9-based engineered dependence systems further reduce escape probability by introducing synthetic dependence of essential genes within the host genome (Moon, 2024).

The quantitative predictive model for escape frequency can be written as:


$$P_{\text{escape}}=1-\prod_{i=1}^{n}{\left(1-p_{i}\right)}^{N_{e}t}$$

Where *n* denotes the number of independent containment layers, *p_i_* the per-site inactivation probability of the i-th containment layer, *N_e_* the effective population size, and *t* the number of strain iterations. Under specified values of *n* = 3, *p_i_* = 10^-8^, *N_e_* = 10^10^, *t* = 10^3^, the P_escape_≈3×10^-13^, the calculated escape frequency satisfies safety thresholds for field release.

Nevertheless, it must be pointed out that the above calculation is based on three strong assumptions, and its practical applicability needs to be carefully evaluated. First, the inactivation mutation rate of each containment layer (*p_i_*) is assumed to be on the order of 10^−8^, whereas the actual value may differ by several orders of magnitude depending on the mechanism—point mutations, IS element insertions, and homologous recombination-mediated deletions range from 10^−6^ to 10^−10^. Studies by Hirota et al. showed that for phosphite-dependent containment strategies, the escape frequency is approximately 10^−4^ to 10^−7^, varying with the escape mechanism (Hirota et al., 2017). The review by Arnolds et al. noted that even the best laboratory containment strategies often have escape frequencies far higher than 10^−8^ under environmental conditions (Arnolds et al., 2021). Second, the effective population size *Ne*​ is not constant in soil—the spatial structure of bacterial populations in soil microhabitats means that *Ne*​ is far lower than the total cell number and fluctuates drastically with moisture, temperature, and nutrient conditions. Third, and most critically, the formula assumes that the containment layers are completely independent; however, if two layers share the same regulatory pathway or the same promoter, a single mutational event may bypass both layers simultaneously, and the formula would severely underestimate the actual escape probability. This equation therefore currently provides a conservative upper-bound estimate rather than a precise predictive tool. Future calibration requires deep mutational scanning experiments to measure empirical mutation spectra combined with spatially explicit population models. Arbel-Groissman et al. further proposed a computational framework to quantify genetic stability of biocontainment circuits (Arbel-Groissman et al., 2023), supplying theoretical tools for accurate escape-frequency prediction.

**Table 5** systematically maps current biocontainment strategies against the MROD principle from the perspective of quantitative design.

**Table 5:** Quantitative design principles for biological containment strategies and the MROD framework.

Design principle	Mechanistic basis	Single-layer escape frequency	MROD implementation
Non-canonical amino acid dependence	Essential enzyme redesign (Mandell et al., 2015)	<10^-^¹^0^	Orthogonal aaRS/tRNA pair
Orthogonal ribosome isolation	Orthogonal rRNA-mRNA pairing (Jia et al., 2017)	<10^-8^	Engineered 16S rRNA
Toxin-antitoxin system	Conditional lethal protein (Halvorsen et al., 2022)	10^-6^-10^-8^	Multiple TA redundancy
Auxotrophy	Essential metabolite dependence (Whitford et al., 2018)	10^-6^-10^-8^	Multi-gene cooperative defects
Intein temperature switch	Temperature-responsive splicing (Foo et al., 2024)	<10^-8^	Multiple temperature-node monitoring
Three-layer MROD combination	Orthogonal mechanisms stacked	<10^-^¹² (predicted)	Multiple redundant orthogonal dependence on essential genes

Single-layer escape frequencies are literature-reported values; the three-layer MROD value is a model prediction not yet validated by field experiments.

### 5.4 Global landscape and core gaps of regulatory frameworks

Field release of genetically engineered PSB involves multi-tiered regulatory requirements. The European Union adopts a process-based regulatory model. Under Directive 2001/18/EC, it implements case-by-case approval for genetically modified microorganisms, with an approval cycle typically ranging from 5 to 8 years. Environmental risk assessment focuses on molecular features of genetic modification, biological traits of the recipient organism, the intended release environment, and interactions between modified organisms and the environment. EFSA updated its risk assessment guidelines for genetically modified microorganisms in 2011, yet progress on dedicated criteria for live engineered microorganisms has been slow. In 2020, EFSA released further guidance on risk assessment for genetically modified animals and food/feed, but dedicated standards for live microorganisms remain at the framework stage (Committee et al., 2020).

The United States follows a product-based regulatory model. The EPA administers oversight under the Microbial Commercial Activity Notice (MCAN) system of the 《Toxic Substances Control Act (TSCA) 》, with a typical review period of 1–2 years. Regulation centers on properties of the final product rather than the modification process (Hoffmann et al., 2023).

China operates under the framework of the《Regulations on Safety Administration of Agricultural Genetically Modified Organisms》and the《Biosafety Law》, implementing graded and phased management for agricultural genetically modified microorganisms. The safety evaluation cycle generally lasts 3–5 years (Bohua et al., 2023). The 《Biosafety Law》, enforced in 2021, provides an overarching legal framework for synthetic biology research. Between 2023 and 2025, China continued refining synthetic biology safety standards, including supporting documents such as the 《Guidelines for Safety Assessment of Synthetic Biology》. Nevertheless, dedicated technical standards governing the release of live engineered microorganisms are still under development (Li et al., 2021b; Lou et al., 2023). The Cartagena Protocol on Biosafety supplies the international legal framework for transboundary movement of genetically modified organisms (Mendelssohn et al., 2003).

The three major regulatory systems represented by China, the EU and the US exhibit fundamental divergences in regulatory philosophy, risk assessment methodologies and approval efficiency, which directly creates institutional barriers to international collaboration.

**Table 6** systematically compares the institutional characteristics of these three regulatory systems.

**Table 6:** Comparison of three global regulatory frameworks for genetically engineered microorganisms.

Dimension	China	European Union	United States
Regulatory philosophy	Risk prevention + tiered management	Process-oriented + precautionary principle	Product-oriented + substantial equivalence
Core legislation	Regulations on Administration of Agricultural GMOs; Biosafety Law	Directive 2001/18/EC	TSCA MCAN regime
Approval timeline	3–5 years	5–8 years	1–2 years
Risk assessment focus	Environmental and food safety	Molecular features + environmental interactions	Product characteristics + intended use
Dedicated guidelines for live microorganisms	Partially established	Framework-level only	Lacking
Recent progress	2023–2025 supporting standards	EFSA 2020 guideline update	No major updates
References	(Li et al., 2021b; Bohua et al., 2023; Lou et al., 2023)	(Committee et al., 2020; Hoffmann et al., 2023)	(Craig et al., 2008; Hoffmann et al., 2023)

Nevertheless, existing regulatory frameworks have a core limitation: most are built upon regulatory experience with “transgenic crops” and lack dedicated risk-assessment criteria tailored to live engineered microorganisms. The fundamental distinction between engineered microbes and transgenic crops lies in the capacity of the former for self-replication, dispersal and evolution. Their risk assessment must address unique questions absent from transgenic crop evaluation, such as HGT of engineered genes within microbial communities, and the long-term fate and evolutionary trajectories of engineered strains in the environment (Hoffmann et al., 2023). Arnolds et al. noted that in the bioeconomy era, progress must be advanced simultaneously along two dimensions: “technical feasibility” and “safety-by-design” (Arnolds et al., 2021). Environmental risk assessment for engineered microbe release should include quantitative estimation of HGT frequency, evaluation of niche replacement potential, and prediction of long-term evolutionary trajectories (Hoy, 2000; Craig et al., 2008). The “safety-by-design” concept proposed by Hoffmann et al. offers a new paradigm for biosafety governance in the synthetic genomics era (Hoffmann et al., 2023).

From the perspective of international collaboration, three structural barriers currently exist. First, lack of mutual recognition of regulatory standards: the three major regulatory systems lack mutual-recognition mechanisms for risk-assessment methodologies, data requirements and safety thresholds. This forces costly repeated safety assessments for the same strain across different jurisdictions, forming a core bottleneck for the cross-border commercialization of engineered strains. Second, weak data-sharing mechanisms: field release and ecological monitoring data from different countries remain isolated in “information silos”, with no unified international database or sharing platform (Committee et al., 2020). Third, North–South capacity gaps: developing countries exhibit substantial disparities from developed nations in risk-assessment technical capacity, regulatory infrastructure and trained professional workforce, which may hinder equitable global access to engineered microbial technologies. A review by Tamang et al. on regulatory frameworks for genetically modified microorganisms further emphasizes the urgency of international harmonization (Tamang et al., 2014). Sun et al. analyzed challenges and advances in biosafety governance in the synthetic biology era, pointing out that China has made remarkable progress in constructing its biosafety legal system, yet international coordination remains a weak link (Sun et al., 2022). This paper proposes establishing a unified risk-tiered international regulatory framework centered on the safety-by-design features of engineered microorganisms. Quantitative predictive models for escape frequency should be incorporated into safety assessment criteria to upgrade safety evaluation from “empirical judgment” to “computable risk quantification”.

### 5.5 Safety as an embedded design parameter

One of the core arguments of this paper is that environmental safety is not an “end-of-engineering checklist item”, but a design parameter embedded at every decision node from target selection through field release. At the target selection stage, the HGT risk of candidate targets should be considered; certain genes carry markedly higher HGT risks and should be eliminated at this stage. During engineering strategy design, genomic integration should be prioritized over plasmid-based strategies to reduce HGT risk, and antibiotic resistance markers should be avoided. At the application-performance evaluation stage, concurrent monitoring of impacts of engineered strains on soil microbial communities is required. Frontloading safety considerations is not a restriction on engineering innovation; rather, it is a prerequisite for ensuring sustainable engineering innovation.

## 6 Four-dimensional mutual feedback: a unified decision framework

### 6.1 Systematic presentation of mutual feedback relationships: twelve bidirectional feedback loops

Based on the systematic analysis of the four preceding dimensions, twelve bidirectional feedback relationships (F1–F12) exist among the four dimensions — gene target(D1), engineering strategy(D2), application performance (D3), and environmental safety(D4) — forming a complete nonlinear decision network. The twelve relationships are organized below into six bidirectional pairs. In the **Supplementary Materials**, we present a four-dimensional coding analysis based on 164 cited references to quantify the frequency of each feedback relationship in the literature (**Supplementary Table S3**; **Supplementary Figure S1**), using empirical data to support the argument for this feedback framework.

Group 1: Feedback between D1 and D2 (feedback density: low; 2 papers coded for both D1 and D2).

F1: Target-to-strategy (D1-to-D2). Molecular properties of target genes — essentiality, operon architecture, and regulatory mode — define the available space of engineering strategies. Essential genes cannot be knocked out and may only be overexpressed or regulated; polycistronic operons are suitable for heterologous expression or genomic integration; phosphorus-starvation-regulated genes can be induced via the *PhoR/PhoB* system. This constraint is best illustrated by engineering of the *pqq* operon: overexpression of individual *pqqA* or *pqqC* alone fails to produce functional gains; coordinated expression of the full operon must be considered (Elena et al., 2014).

F2: Strategy-to-target (D2-to-D1). Results from engineering experiments deepen understanding of target gene function. CRISPR knockout experiments directly reveal gene essentiality for phosphate solubilization, while overexpression experiments uncover nonlinear relationships between gene dosage and functional output. For instance, overexpression experiments in *H. seropedicae* Z67 by Wagh et al. demonstrated synergistic enhancement between *pqq* and *gad* genes (Wagh et al., 2016), whereas systematic screening by Shulse et al. revealed functional differences among phytase genes in rhizosphere-colonizing bacteria (Shulse et al., 2019).

Group 2: Feedback between D2 and D3 (feedback density: moderate; 4 papers coded for both D2 and D3).

F3: Strategy-to-performance (D2-to-D3). Selection of engineering strategy directly determines field application performance. Genomic integration offers superior genetic stability relative to plasmid systems, yet may yield lower functional output due to copy-number limitations. Plasmid systems deliver higher short-term functional output but undergo faster functional decay under field conditions (Andreessen et al., 2014). CRISPR-edited strains possess advantages in genetic stability, yet positional effects at genomic integration sites may alter expression levels.

F4: Performance-to-strategy (D3-to-D2). Field performance data serve as the ultimate judge of engineering strategies. Poor rhizosphere colonization or insufficient sustained functional expression directly feeds back to revise engineering strategies — switching from plasmid systems to genomic integration, or from single-gene overexpression toward multi-gene coordinated optimization. The soil-microcosm ALE pre-adaptation strategy proposed herein is a concrete implementation of F4 feedback: after field performance data reveal decay patterns, directed evolution is performed under simulated field conditions in microcosms containing target soil, translating “failure” into “revision” (Lapanse et al., 2022).

Group 3: Feedback between D3 and D4 (feedback density: highest; 9 papers coded for both D3 and D4).

F5: Performance-to-safety (D3-to-D4). Field application performance reflects not only strain function but also safety risks. Strains with robust colonization exhibit strong functional performance, yet their long-term persistence also implies greater risk of ecological perturbation. Strains with sustained functional expression maintain engineered genes in the environment for longer periods, widening the window of opportunity for HGT (Babalola, 2014).

F6: Safety-to-performance (D4-to-D3). Safety design may affect application performance. Biological containment strategies (e.g., auxotrophy) can reduce strain competitiveness in the rhizosphere, and accidental activation of kill switches may abolish function. Trade-offs exist between safety design and functional optimization, requiring global optimization rather than local maximization (Halvorsen et al., 2022).

Group 4: Feedback between D4 and D2 (feedback density: moderate; 7 papers coded for both D2 and D4).

F7: Safety-to-strategy (D4-to-D2). Safety considerations constrain the available engineering strategy space. Broad-host-range plasmids facilitate genetic manipulation yet carry high HGT risk and are unsuitable for constructing strains intended for field release. Antibiotic resistance markers simplify screening but are unacceptable for field deployment (Hoffmann et al., 2023). These constraints force engineering strategy design to shift from “maximizing operational convenience” toward “optimizing safety”.

F8: Strategy-to-safety (D2-to-D4). The choice of engineering strategy directly defines the spectrum of safety hazards. Plasmid-based strategies require stricter containment measures due to high HGT risk, while genomic integration permits relatively simplified containment design owing to lower HGT risk. Off-target effects from CRISPR editing may introduce unanticipated safety risks (Huang et al., 2022). Heterologous expression strategies introduce exogenous gene fragments with unknown HGT potential.

Group 5: Feedback between D1 and D3 (feedback density: very low; 2 papers coded for both D1 and D3).

F9: Target-to-performance (D1-to-D3). Target selection preconditions the prospects of application performance. Selecting gcd as a target may boost inorganic phosphorus solubilization, yet if the target soil is dominated by organic phosphorus, this choice inherently caps the performance ceiling. Targeting regulatory genes such as PhoR/PhoB may enable coordinated multi-gene regulation, yet the complexity of regulatory networks increases uncertainty in performance prediction (Santos-Beneit et al., 2016).

F10: Performance-to-target (D3-to-D1). Field performance data establish empirical priorities for target selection. Target genes with consistent performance across multiple crops and soil types deserve higher priority. Discrepancies between field performance data and laboratory molecular mechanistic studies may uncover novel regulatory nodes or functional genes (Imam et al., 2021).

Group 6: Feedback between D1 and D4 (feedback density: low; 4 papers coded for both D1 and D4).

F11: Target-to-safety (D1-to-D4). Selection of target genes shapes the safety risk spectrum. Certain target genes (e.g., tra genes involved in plasmid transfer, antibiotic resistance genes) should not be used as engineering targets despite their functional importance, as their modification may elevate HGT risk. The conservation level of target genes also influences ecological consequences after HGT; highly conserved genes are already widespread within indigenous communities, rendering their HGT risks relatively manageable.

F12: Safety-to-target (D4-to-D1). Safety risks may alter the priority of target selection. Safety-assessment data such as HGT frequency and ecological disturbance magnitude can guide a “safety priority ranking” for target selection. Targets carrying high safety risks should be excluded or demoted even when they yield substantial functional gains (Hoffmann et al., 2023).

### 6.2 From linear thinking to systems thinking

The twelve feedback relationships described above lead to a fundamental conclusion: the success of PSB genetic engineering does not hinge on breakthroughs in any single dimension, but on coordinated optimization across all four dimensions. Conventional linear thinking — “select targets first, design strategies next, validate performance, and assess safety last” — ignores cross-dimensional feedback. This may yield engineered strains with outstanding performance in one dimension but fatal flaws in others.

Systems thinking requires treating the four dimensions as an integrated whole and incorporating feedback from the other three dimensions at every decision node. For example, at the target-selection stage, researchers must consider not only functional importance of the target but also engineering tractability (reverse of F2), impacts on application performance (F9–F10), and safety risks of the target (F11–F12).

### 6.3 Current core bottlenecks: three modes of broken feedback and representative cases

The core bottleneck in this field is not technological deficiency in a single dimension, but “broken feedback” between dimensions. Three modes of breakdown are identified, each supported by representative research cases.

Breakdown Mode 1: Target–strategy disconnection. Most studies select targets without considering engineering strategy feasibility, and choose strategies without analyzing constraints imposed by target characteristics. A typical example is the large body of overexpression studies targeting single genes *pqqA* or *pqqC*. Although these works report improved phosphate-solubilizing activity, failure to account for multi-gene coordination within the *pqq* operon means functional gains often fall far below the potential ceiling achievable by engineering the full operon (Elena et al., 2014). Another representative case is the series of experiments by Wagh et al.: in *H. seropedicae* Z67, expression of *pqq* alone yielded 23.5 mM gluconate, while combined expression of *pqq* and *gad* achieved phosphate-solubilizing activity of 457.7 mM. This progressive result itself demonstrates the cost of decoupling target selection from strategy design (Wagh et al., 2014; Wagh et al., 2016). In the literature-coding data from **Supplementary Materials**, only 2 papers show D1–D2 feedback density (1.2% of the 164 cited references), quantitatively confirming the prevalence of this breakdown.

Breakdown Mode 2: Strategy–performance disconnection. Functional validation of most engineered strains remains confined to laboratory shake flasks and greenhouse pot trials, lacking systematic data on sustained functional expression under field conditions. Optimization of engineering strategies targets “functional maximization under laboratory conditions”, rather than “functional sustainability under field conditions”. A hallmark example is the phytase-engineered strain study by Shulse et al.: remarkable growth promotion was achieved in *Arabidopsis thaliana* with phytate as the sole phosphorus source, representing landmark synthetic biology work in PSB engineering (Shulse et al., 2019). However, this study lacked field validation under natural soil conditions, and the real-world performance of the engineered strain in complex microbiomes and heterogeneous phosphorus sources remains unknown. Another representative case is the wheat field trial by Meyer et al.: *P. protegens* CHA0 exhibited excellent phosphate-solubilizing activity in the laboratory yet failed to significantly enhance wheat uptake of ³³P-labeled phosphorus under field conditions (Meyer et al., 2019). This “laboratory success, field failure” outcome typifies strategy–performance disconnection. According to supplementary coding data, D2–D3 feedback density is 4 papers (2.4%). While higher than D1–D2, it remains low.

Breakdown Mode 3: Safety full-chain disconnection. In most studies, safety assessment is an “afterthought” rather than an “embedded upfront consideration”. Target selection ignores safety risks, engineering strategy design omits containment requirements, and field performance validation is not paired with concurrent monitoring of ecological effects. Supplementary coding data show D1–D4 feedback density at 4 papers and D2–D4 at 7 papers. Although relatively higher than other feedback pairs, the D1–D4 and D2–D4 literature mainly cite target- or strategy-related papers within safety discussions, rather than original studies on target selection and strategy design that actively embed safety considerations. The consequences of this breakdown are severe: even if engineered strains perform well in laboratory and greenhouse settings, missing safety assessment data prevents regulatory approval and commercialization. All three international regulatory systems (China, EU, US) require environmental safety data for approval, and safety full-chain disconnection means most engineered strains are ineligible to enter regulatory pipelines from the outset (Committee et al., 2020; Hoffmann et al., 2023).

## 7 Future trends: from single-gene manipulation to system-level design

### 7.1 Multi-omics-driven rational design

Rapid advances in multi-omics technologies provide unprecedented datasets for rational design of PSB. Genome-scale metabolic models (GEMs) can predict the impacts of genetic manipulation on metabolic networks at the systems level. Reconstruction and analysis of genome-scale metabolic networks in *Pseudomonas fluorescens* uncovered the central carbon metabolic regulatory network of this strain (Huang and Lin, 2019). Genome characterization and reconstruction of the complete low-phosphorus stress response system in *Escherichia coli* HRY1 offer a systems-level perspective for understanding phosphorus metabolism in this strain (He et al., 2026). GEMs have been widely applied in synthetic biology and metabolic engineering to guide strain design (Karlsen et al., 2018; Nogales et al., 2019).

Deep learning for enzyme kinetic prediction supplies new tools for metabolic engineering. The DLTKcat model developed by Qiu et al. predicts temperature-dependent enzyme turnover rates from protein sequences (Qiu et al., 2023), a capability critical for simulating phosphate-solubilizing enzyme output across variable environmental temperatures. Integrating such predictive tools with GEMs holds promise for forecasting metabolic function of engineered PSB under field conditions. Metabolic modeling under multi-dimensional constraints — integrating thermodynamic feasibility, enzyme kinetics and omics data — is emerging as a core technology for next-generation strain design (Li et al., 2018; Yang et al., 2018).

### 7.2 Synthetic microbial communities: from single strains to multi-species coordination

Synthetic microbial communities (SynComs) represent a frontier in microbiome engineering in recent years. For agricultural applications, SynComs have demonstrated substantial growth-promoting potential. De la Vega-Camarillo et al. assembled three SynComs using PGPB isolated from the Jala maize cultivar; in field trials, maize yield rose from 11 t/ha to 16 t/ha, while rust incidence dropped from 12.5% to 8% (Vega-Camarillo et al., 2023). A recent review by Cui et al. comprehensively summarized strategies for SynComs to enhance soil health and crop yield in sustainable agriculture (Cui et al., 2025). Wang et al. systematically traced the development of SynComs from theoretical exploration to applied innovation, noting that integration of ecological principles and computational tools is central to rational SynCom design (Wang et al., 2026). Bashizi et al. demonstrated SynCom application to boost pepper resistance against *Phytophthora capsici*, with markedly lower disease incidence in SynCom treatments than in single-strain treatments (Bashizi et al., 2025). O’Callaghan and Shi reported SynComs as bioinoculants to overcome rice production limitations in acidic soils (O’Callaghan and Shi, 2024).

For PSB engineering, SynCom strategies carry unique advantages. Distinct phosphate-solubilizing functions — inorganic phosphorus solubilization, organic phosphorus mineralization, siderophore-mediated iron–phosphorus release — can be partitioned among different community members, preventing excessive metabolic burden on a single strain tasked with multiple functions. Furthermore, communities containing niche-complementary strains may achieve superior spatiotemporal coverage and functional persistence compared with monocultures (Shayanthan et al., 2022). Nevertheless, interactions among community members are complex and hard to predict; laboratory-assembled communities may disassemble under field environmental shifts. Competition among members during serial passaging can drive drift in community structure, representing a key stability challenge for field deployment of SynComs. Liang et al. proposed that construction of environmental SynComs must be grounded in both engineering and ecological principles (Liang et al., 2022). Shamrick et al. used HGT to dynamically program “division of labor” within communities (Hamrick et al., 2024). By rationally simplifying a sorghum SynCom, Pettinga et al. found functional complementarity rather than member count is key to maintaining growth-promoting performance (Pettinga et al., 2026).

### 7.3 Smart response systems: precise regulation for on-demand expression

Smart response systems achieve on-demand expression of phosphate-solubilizing functions under field conditions by constructing environmental signal-sensing and gene-expression regulatory circuits. Key components for building smart response systems include phosphorus-sensing promoters (e.g., PhoB-dependent promoters), quorum-sensing systems (to detect rhizosphere colonization density), and plant-signal-sensing elements (e.g., root-exudate-responsive promoters). The “molecular plant nutrition” concept proposed by Fernandez et al. provides a theoretical foundation for regulating microbial function using plant signals (Gómez-Merino et al., 2015). Quorum sensing plays a core regulatory role in rhizosphere microbe–plant interactions and can serve as a control node for functional expression in engineered strains (Ortiz-Castro and López-Bucio, 2019).

However, the reliability of smart response systems under field conditions remains to be validated. Signal noise in field environments, degradation and diffusion of signaling molecules, and signal interference from other community members may compromise the precision of sensing circuits. Design principles for genetic circuits in synthetic biology — including modularity, orthogonality and robustness — offer methodological guidance for engineering smart response systems (Rawat et al., 2021; Chiarnanunt and White, 2023).

### 7.4 Quantitative prediction of evolutionary stability and escape frequency: key scientific questions for the next five years

**Figure 3** illustrates the development trajectory of PSB genetic engineering from 2010 to 2025. Looking ahead, quantitative prediction of evolutionary stability and escape frequency represent the most critical scientific questions for the next five years, topics barely addressed in existing reviews.

**Figure 3:**
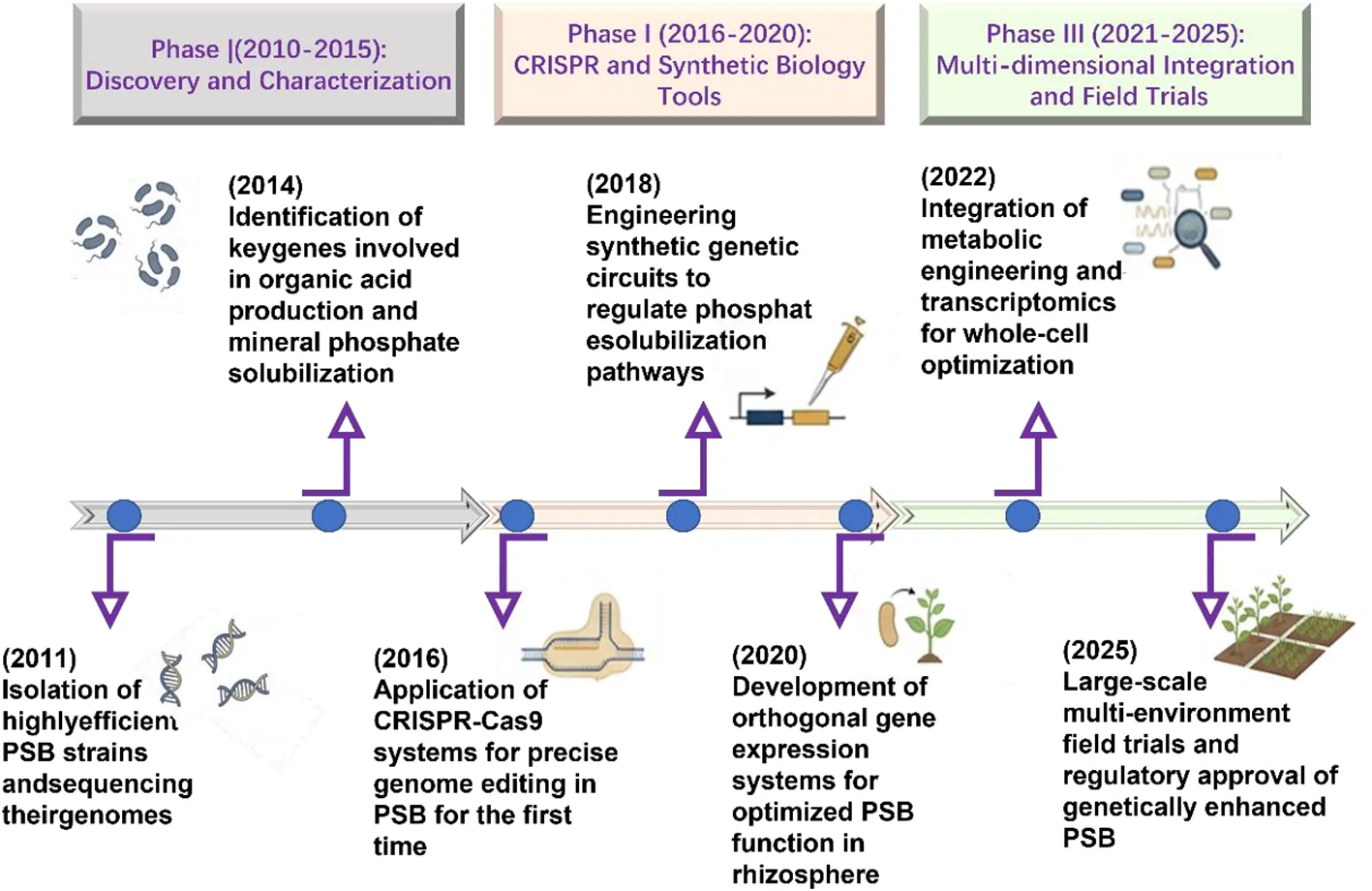
Development trajectory of PSB genetic engineering (2010–2025).

The core issue of evolutionary stability is: to what extent the designed function of engineered strains can resist evolutionary erosion during long-term persistence in the field. Heterologous genes, high expression burden, and artificial regulatory circuits carried by engineered strains may become fitness costs under natural selection, driving reversion mutations, expression silencing, or loss of function (Lehouritis et al., 2017; Barnum et al., 2021). This is a deeper issue beyond traditional discussions of “suicide switches”—even if the containment circuit itself does not fail, the phosphate-solubilizing function of the engineered strain may gradually decay during evolution, causing it to lose application value.

Pathways to address this issue include: (1) At the target selection stage, prioritize gene loci with minimal impact on host fitness, use comparative genomics to identify evolutionarily conserved functional modules, and avoid high-mutation-rate hotspots; (2) At the engineering strategy design stage, introduce “evolutionary buffering” designs—such as multi-copy redundancy of key functional genes, synonymous mutation elimination of IS element recognition sites, and sequence optimization based on mutation prediction models; (3) At the application efficacy assessment stage, incorporate evolutionary stability into the core indicator system, quantifying the functional decay rate through long-term serial passage experiments in microcosms. The integration of ALE experiments and whole-genome sequencing provides an experimental framework for assessing evolutionary stability (Zhang et al., 2019).

Quantitative prediction models of escape frequency need to be upgraded from the current deterministic framework to a probabilistic framework. The model $P_{\text{escape}}=1-\prod_{i=1}^{n}{\left(1-p_{i}\right)}^{N_{e}t}$ proposed in this paper provides a first-principles computational basis for multi-layer containment design, but this model currently relies on multiple assumed parameters (uniformity of *pi*, constancy of *Ne*, and independence among containment layers). Future research directions include: (1) Experimentally determining the actual inactivation mutation spectra and frequency distributions of each containment layer through deep mutational scanning; (2) Incorporating environmental heterogeneity (temperature, pH, and nutrient gradients in soil microhabitats) into spatial variability models of *Ne*​ and *pi*​; (3) Developing machine learning-based escape frequency prediction tools that integrate genomic sequence features, population dynamic parameters, and environmental variables to achieve in silico pre-assessment of engineered strain safety. The computational framework of Arbel-Groissman et al. and the CRISPR-Cas containment systems of Collias et al. provide important starting points for this direction (Arbel-Groissman et al., 2023; Collias et al., 2023).

### 7.5 Forward engineering of safety design

Future safety design will shift from “passive containment” to “forward engineering”—rather than adding containment measures after strain construction is completed, safety features will be embedded into the genetic design of the strain from the target selection stage. The core idea of forward engineering is to make the survival and functional expression of engineered strains depend on artificially designed conditions that do not exist or are unavailable in natural environments.

Orthogonal genetic systems are the core technology of forward engineering. By designing orthogonal ribosome-mRNA pairs, orthogonal aminoacyl-tRNA systems, or orthogonal DNA replication systems, the reading and expression of engineered genes can be made completely dependent on orthogonal translation or replication machinery (Jia et al., 2017). Mandell et al. computationally redesigned essential enzymes to make engineered strains dependent on non-canonical amino acids for survival, achieving escape frequencies below the detection limit under laboratory conditions (Mandell et al., 2015). The maturation of these technologies will fundamentally change the paradigm of safety design for engineered microorganisms. CRISPR-based engineering dependency systems—by introducing synthetic dependency of essential genes into the genome—represent the latest advances in forward engineering (Kato and Mori, 2024; Moon, 2024).

### 7.6 Four-dimensional synergy is a prerequisite for industrial translation

Based on the analysis of the four-dimensional feedback framework, this paper proposes key breakthrough pathways for the next 5 to 10 years. First, systematic validation of field genetic stability is the primary task. It is recommended to select 3 to 5 representative crop-soil systems to monitor the genetic stability and functional persistence of engineered strains across growing seasons, while incorporating the soil microcosm ALE pre-adaptation strategy into standard procedures. Second, the accumulation of long-term ecological monitoring data is the basis for safety assessment. It is recommended to establish a long-term ecological monitoring network for field release of engineered PSB, accumulating longitudinal data spanning at least 3 to 5 years. Third, the establishment of internationally harmonized regulatory standards is the institutional guarantee for industrial translation. It is recommended to establish an internationally harmonized regulatory framework based on risk grading, centered on the “safety by design” characteristics of engineered microorganisms, incorporating quantitative prediction models of escape frequency into safety assessment standards. Fourth, the establishment of the “four-dimensional synergy” methodology is the core of the research paradigm shift. It is recommended to develop four-dimensional synergistic computational decision-support tools that integrate multi-dimensional constraints of target-strategy-efficacy-safety, providing systematic quantitative analysis tools for the design, assessment, and decision-making of engineered strains.

## 8 Conclusion

The core challenge for genetically engineered PSB to move from the laboratory to large-scale field application is not a single technological breakthrough in any one dimension, but rather systematic synergy among four dimensions—gene target, engineering strategy, application efficacy, and environmental safety. By proposing the “four-dimensional reciprocal feedback framework” (4D-RFF), this paper systematically demonstrates twelve types of bidirectional feedback relationships among these dimensions and reveals the core bottleneck in the current field: “feedback disconnection” among dimensions.

The disconnection between target selection and engineering strategy (literature-coded feedback density of only 2 references, 1.2%) leads to engineered strains with excellent function under laboratory conditions but inability to stably inherit in the field. The disconnection between engineering strategy and application efficacy (feedback density of 4 references, 2.4%) results in a large gap between laboratory function and field performance that cannot be bridged through strategy revision. The disconnection between safety assessment and the full chain makes it difficult for engineered strains to obtain regulatory approval and achieve commercialization. These three disconnections together constitute the deep-seated reason for the dilemma of “impressive yield-increase data yet failure to commercialize.”

The core contributions of this paper are: (1) transforming the four dimensions from “independent discussions” into “systematic reciprocal feedback,” proposing a complete framework of twelve types of bidirectional feedback relationships, along with a three-tier decision logic for engineering strategy selection and a multidimensional paradigm for application efficacy assessment; (2) proposing a concrete pathway to convert field failure into strategy-revision feedback (F4) using soil microcosm ALE pre-adaptation, while candidly discussing the limitations of ALE in liquid media, and the “multiple redundancy orthogonal dependency” (MROD) design principle for essential genes, which sets a quantitative target of reducing evolutionary escape frequency to<10^−12^, and identifying three strong assumptions of escape prediction models and their calibration needs; (3) establishing a quantitative prediction model of escape frequency, $P_{\text{escape}}=1-\prod_{i=1}^{n}{\left(1-p_{i}\right)}^{N_{e}t}$, providing a methodological basis for biocontainment design to shift from qualitative enumeration to quantitative design; and (4) diagnosing three modes of feedback disconnection—target–strategy disconnection, strategy–efficacy disconnection, and safety–full-chain disconnection—with typical research cases and quantitative evidence from literature coding provided for each.

Three key research gaps currently exist in this field. First, the lack of long-term ecological effect data—the cross-year ecological effects of engineered PSB after field release have hardly been systematically studied, especially deep ecological risks such as trophic cascades, erosion of functional redundancy, and disturbance of the soil food web. Second, the lack of dedicated risk assessment criteria tailored to living engineered microorganisms—existing regulatory frameworks are mainly based on experience with GM crops, fail to fully consider the self-replication, dispersal, and evolutionary capacities of engineered microorganisms, and the lack of mutual recognition of standards among the three major international regulatory systems constrains international cooperation. Third, theoretical and experimental research on evolutionary stability is severely insufficient—the rate and mechanism of functional decay of engineered strains during long-term field persistence remain unclear, and quantitative prediction models of escape frequency lack experimental calibration, particularly regarding the actual distribution of inactivation mutation rates of each containment layer, the spatiotemporal heterogeneity of soil effective population size, and the quantification of non-independence among containment layers.

The core proposition for the future is to transform four-dimensional reciprocal feedback from an analytical framework into a methodology for engineering practice. This requires multi-omics-driven rational design to provide a data foundation, synthetic microbial community strategies to provide functional division of labor and niche complementarity, smart responsive systems to achieve on-demand expression under field conditions, and forward engineering of safety design to ensure the sustainability of engineering innovation. Quantitative prediction of evolutionary stability and escape frequency will be the key scientific proposition determining whether engineered PSB can truly overcome the bottleneck of translation from the laboratory to the field.

## Data Availability

The original contributions presented in the study are included in the article/**Supplementary Material**. Further inquiries can be directed to the corresponding author.
