# Supplementary material for: Decision pathways for genetically modified phosphate-solubilizing bacteria: a four-dimensional synergistic decision-making framework from genetic targets to field deployment
Source: Front Plant Sci. 2026 Oct 28;17:1949787. doi: 10.3389/fpls.2026.1949787 (PMC13751954; doi:10.3389/fpls.2026.1949787)
Supplement: Supplementary file 1 [file SupplementaryFile1.docx]

# Supplementary Materials: Literature Coding Statistics and Cross-Dimension Interaction Density Heatmap

## Table S1: Overview of Literature Coding Statistics

| Metric | Value |
| --- | --- |
| Total papers in library | 392 |
| Total cited papers in manuscript | 163 |
| Coded papers (appearing in ≥1 dimension subsection) | 153 |
| Uncoded papers (abstract/introduction/discussion only) | 10 |
| Single-dimension coded | 131 |
| Multi-dimension coded | 22 |
| Two-dimension coded | 19 |
| Three-dimension coded | 3 |
| Four-dimension fully coded | 0 |

## Figure S1: Cross-Dimension Interaction Density Heatmap

Interaction density is defined as the number of papers cited in both dimension-i and dimension-j subsections. Diagonal cells are grayed out. Deeper red indicates higher interaction density (white = 0 papers, dark red = 9 papers).

## Table S2-1: Gene Targets (Molecular Mechanisms and Editable Nodes) — Coded Papers: 34 (22.2% of total)

| Citation Key | Dims | Title |
| --- | --- | --- |
| andrewlneal2017 | D1 | Phylogenetic distribution, biogeography and the effects of land management upon bacterial non-specific Acid phosphatase ... |
| benjesuorsemwenenagb2020 | D1 | Unveiling Plant-Beneficial Function as Seen in Bacteria Genes from Termite Mound Soil |
| claudiaelena2014 | D1/D2/D4 | Expression of codon optimized genes in microbial systems: current industrial applications and perspectives |
| dehongzheng2018 | D1 | Activation of PhoBR under phosphate-rich conditions reduces the virulence of Xanthomonas oryzae pv. oryzae |
| deniselüttmann2012 | D1 | The phosphotransferase protein EIIANtr modulates the phosphate starvation response through interaction with histidine ki... |
| fernandosantosbeneit2017 | D1/D4 | Glycopeptide resistance: Links with inorganic phosphate metabolism and cell envelope stress |
| franciscoxnascimento2021 | D1 | Genomic Analysis of the 1-Aminocyclopropane-1-Carboxylate Deaminase-Producing Pseudomonas thivervalensis SC5 Reveals Its... |
| fujingpan2024 | D1 | Vegetation recovery reshapes the composition and enhances the network connectivity of phoD -harboring microorganisms to ... |
| huiwan2017 | D1 | Identification of transporter proteins for PQQ-secretion pathways by transcriptomics and proteomics analysis in Gluconob... |
| huoqinghuang2009 | D1 | Diversity of Beta-Propeller Phytase Genes in the Intestinal Contents of Grass Carp Provides Insight into the Release of ... |
| ilhemsaadouli2021 | D1 | The Impact of the Inoculation of Phosphate-Solubilizing Bacteria Pantoea agglomerans on Phosphorus Availability and Bact... |
| jitendrawagh2014 | D1/D2/D4 | Heterologous expression of pyrroloquinoline quinone (pqq) gene cluster confers mineral phosphate solubilization ability ... |
| kellybotero2018 | D1 | A genome-scale metabolic model of potato late blight suggests a photosynthesis suppression mechanism |
| licunzhang2023 | D1 | Land reclamation increased organic P fractions and phosphatase activities, and strengthened the co-occurrence networks o... |
| linlinwang2022 | D1 | Genomic Analysis of Pseudomonas asiatica JP233: An Efficient Phosphate-Solubilizing Bacterium |
| lolowalmarzan2011 | D1/D3 | Metabolic regulation of Escherichia coli and its phoB and phoR genes knockout mutants under phosphate and nitrogen limit... |
| muhammadkashifhanif2015 | D1 | Isolation and characterization of a β-propeller gene containing phosphobacterium Bacillus subtilis strain KPS-11 for gro... |
| nataliastosiek2019 | D1 | Carbon-Phosphorus Lyase—the State of the Art |
| peichunlisahsu2015 | D1 | hemX is required for production of 2‐ketogluconate, the predominant organic anion required for inorganic phosphate solub... |
| prassanchoudhary2022 | D1 | Understanding the biological role of PqqB in Pseudomonas stutzeri using molecular dynamics simulation approach |
| rajinderkaur2021 | D1 | Molecular and physiological manipulations in rhizospheric bacteria |
| ranan2016 | D1 | Regulation of Pyrroloquinoline Quinone-Dependent Glucose Dehydrogenase Activity in the Model Rhizosphere-Dwelling Bacter... |
| sabinearagot2015 | D1 | phoD Alkaline Phosphatase Gene Diversity in Soil |
| sabinearagot2016 | D1/D3 | Soil phoD and phoX alkaline phosphatase gene diversity responds to multiple environmental factors |
| samikshajoshi2023 | D1 | Functional characterization and molecular fingerprinting of potential phosphate solubilizing bacterial candidates from S... |
| scaria2010 | D1 | Crystallization and preliminary X-ray characterization of a catalytic and ATP-binding domain of a putative PhoR histidin... |
| tandradfraser2015 | D1 | Soil bacterial phoD gene abundance and expression in response to applied phosphorus and long-term management |
| wenjiewan2020 | D1 | Isolation and Characterization of Phosphorus Solubilizing Bacteria With Multiple Phosphorus Sources Utilizing Capability... |
| wenzhu2020 | D1 | Biogenesis of the peptide-derived redox cofactor pyrroloquinoline quinone |
| xiaodongchen2021 | D1 | Impact of grassland afforestation with contrasting tree species on soil phosphorus fractions and alkaline phosphatase ge... |
| xiaoyanhuang2019 | D1/D4 | Reconstruction and analysis of a three-compartment genome-scale metabolic model for Pseudomonas fluorescens |
| yajunhu2018 | D1 | Effects of long-term fertilization on phoD -harboring bacterial community in Karst soils |
| yaoqingshen2012 | D1 | Distribution and Properties of the Genes Encoding the Biosynthesis of the Bacterial Cofactor, Pyrroloquinoline Quinone |
| yuhuizhang2018 | D1 | GlnR positive transcriptional regulation of the phosphate-specific transport system pstSCAB in Amycolatopsis mediterrane... |

## Table S2-2: Engineering Strategies (Expression Systems, Gene Editing, and Metabolic Engineering) — Coded Papers: 32 (20.9% of total)

| Citation Key | Dims | Title |
| --- | --- | --- |
| aadishrawat2021 | D2/D4 | Cysteine proteases: Battling pathogenic parasitic protozoans with omnipresent enzymes |
| aditibuch2010 | D2 | Heterologous expression of phosphoenolpyruvate carboxylase enhances the phosphate solubilizing ability of fluorescent ps... |
| alexanderdasilvavale2022 | D2 | Perspective on the use of synthetic biology in rudimentary food fermentations |
| allisonsharrar2024 | D2 | Discovery and engineering of AiEvo2, a novel Cas12a nuclease for human gene editing applications |
| bijoyapaul2020 | D2 | CRISPR-Cas12a: Functional overview and applications |
| björnandreeßen2014 | D2/D3/D4 | Influence of the operon structure on poly(3-hydroxypropionate) synthesis in Shimwellia blattae |
| christinenshulse2019 | D2/D4 | Engineered Root Bacteria Release Plant-Available Phosphate from Phytate |
| claudiaelena2014 | D1/D2/D4 | Expression of codon optimized genes in microbial systems: current industrial applications and perspectives |
| guillermofernandezbu2022 | D2/D3 | Novel Production Methods of Polyhydroxyalkanoates and Their Innovative Uses in Biomedicine and Industry |
| igordanielalvesribei2020 | D2 | Use of Mineral Weathering Bacteria to Enhance Nutrient Availability in Crops: A Review |
| ivanmijakovic2005 | D2 | Tunable promoters in systems biology |
| jianli2021 | D2 | Microbial-Derived Polyhydroxyalkanoate-Based Scaffolds for Bone Tissue Engineering: Biosynthesis, Properties, and Perspe... |
| jitendrawagh2014 | D1/D2/D4 | Heterologous expression of pyrroloquinoline quinone (pqq) gene cluster confers mineral phosphate solubilization ability ... |
| jitendrawagh2016 | D2/D4 | Inoculation of genetically modified endophytic Herbaspirillum seropedicae Z67 endowed with gluconic and 2-ketogluconic a... |
| joshuarelmore2023 | D2 | High-throughput genetic engineering of nonmodel and undomesticated bacteria via iterative site-specific genome integrati... |
| junhuang2022 | D2/D4 | CRISPR-Cas12a induced DNA double-strand breaks are repaired by multiple pathways with different mutation profiles in Mag... |
| justintan2020 | D2/D3 | Independent component analysis of E. coli 's transcriptome reveals the cellular processes that respond to heterologous g... |
| laurenceyang2018 | D2/D3 | Modeling the multi-scale mechanisms of macromolecular resource allocation |
| liqunjin2019 | D2 | Promoter engineering strategies for the overproduction of valuable metabolites in microbes |
| meiyiyan2017 | D2 | CRISPR-Cas12a-Assisted Recombineering in Bacteria |
| meliawatimeliawati2021 | D2 | Recent advances of Cas12a applications in bacteria |
| michellejchua2022 | D2 | Rapid, Efficient, and Cost-Effective Gene Editing of Enterococcus faecium with CRISPR-Cas12a |
| mingzhao2019 | D2 | Engineering diverse eubacteria promoters for robust Gene expression in Streptomyces lividans |
| monisatharkhan2026 | D2 | Targeted genome editing of the non-model cyanobacterium Cyanothece PCC 7425 via CRISPR/Cas12a |
| ningxu2019 | D2 | Recent advances in the applications of promoter engineering for the optimization of metabolite biosynthesis |
| sarabaldanta2022 | D2 | SEVA-Cpf1, a CRISPR-Cas12a vector for genome editing in cyanobacteria |
| weimeng2023 | D2 | Construction and application of a new CRISPR/Cas12a system in Stenotrophomonas AGS-1 from aerobic granular sludge |
| xiangao2018 | D2 | A Multiplex Genome Editing Method for Escherichia coli Based on CRISPR-Cas12a |
| xiaofanfeng2021 | D2 | Saccharomyces cerevisiae Promoter Engineering before and during the Synthetic Biology Era |
| xuemihao2023 | D2 | CRISPR/Cas12a-Mediated Genome Editing in Thioalkalivibrio versutus |
| xuzhang2020 | D2 | Synthetic Biology and Genome-Editing Tools for Improving PHA Metabolic Engineering |
| yujiezhang2025 | D2 | Establishment of a RecET-Assisted CRISPR-Cas12a System for Large Deoxyribonucleic Acid-Fragment Manipulation in Zymomona... |

## Table S2-3: Application Efficacy (Laboratory-to-Field Translation) — Coded Papers: 44 (28.8% of total)

| Citation Key | Dims | Title |
| --- | --- | --- |
| adrianaambrosini2015 | D3/D4 | Ecological role of bacterial inoculants and their potential impact on soil microbial diversity |
| alainajlapanse2022 | D3/D4 | Adaptive laboratory evolution for increased temperature tolerance of the diatom Nitzschia inconspicua |
| amandineducoussodétr2022 | D3 | Diversity of Phosphate Chemical Forms in Soils and Their Contributions on Soil Microbial Community Structure Changes |
| annatimofeeva2022 | D3 | Prospects for Using Phosphate-Solubilizing Microorganisms as Natural Fertilizers in Agriculture |
| asfarizvi2021 | D3 | Psychrophilic Bacterial Phosphate-Biofertilizers: A Novel Extremophile for Sustainable Crop Production under Cold Enviro... |
| birgitescharf2016 | D3 | Chemotaxis signaling systems in model beneficial plant–bacteria associations |
| björnandreeßen2014 | D2/D3/D4 | Influence of the operon structure on poly(3-hydroxypropionate) synthesis in Shimwellia blattae |
| brianarenda2014 | D3 | Genome Instability Mediates the Loss of Key Traits by Acinetobacter baylyi ADP1 during Laboratory Evolution |
| dmitryderyabin2019 | D3 | Plant-Derived Inhibitors of AHL-Mediated Quorum Sensing in Bacteria: Modes of Action |
| dorothébelotte2007 | D3 | An experimental test of local adaptation in soil bacteria |
| esaúdelavegacamarill2023 | D3/D4 | Promotion of the growth and yield of Zea mays by synthetic microbial communities from Jala maize |
| esmaeilbakhshandeh2014 | D3 | Phosphate solubilization potential and modeling of stress tolerance of rhizobacteria from rice paddy soil in northern Ir... |
| estherblancoromero2023 | D3 | Adaption of Pseudomonas ogarae F113 to the Rhizosphere Environment—The AmrZ-FleQ Hub |
| gmeyer2019 | D3/D4 | Pseudomonas protegens CHA0 does not increase phosphorus uptake from 33 P labeled synthetic hydroxyapatite by wheat grown... |
| guillermofernandezbu2022 | D2/D3 | Novel Production Methods of Polyhydroxyalkanoates and Their Innovative Uses in Biomedicine and Industry |
| gustavosantoyo2021 | D3 | Rhizosphere Colonization Determinants by Plant Growth-Promoting Rhizobacteria (PGPR) |
| hyadav2013 | D3 | Optimization of culture conditions for phosphate solubilization by a thermo-tolerant phosphate-solubilizing bacterium Br... |
| idaromano2020 | D3 | Effectiveness of Plant Beneficial Microbes: Overview of the Methodological Approaches for the Assessment of Root Coloniz... |
| israelikoyi2024 | D3 | Manipulation of sward diversity is a more effective management strategy than addition of microbial inoculants in intensi... |
| jacquelinemchaparro2012 | D3/D4 | Manipulating the soil microbiome to increase soil health and plant fertility |
| jessicaaparecidaferr2023 | D3 | Meta-omics integration approach reveals the effect of soil native microbiome diversity in the performance of inoculant A... |
| justintan2020 | D2/D3 | Independent component analysis of E. coli 's transcriptome reveals the cellular processes that respond to heterologous g... |
| katilavarivarn2013 | D3 | Colonization strategies of Pseudomonas fluorescens Pf0-1: activation of soil-specific genes important for diverse and sp... |
| kezhao2014 | D3 | Maize rhizosphere in Sichuan, China, hosts plant growth promoting Burkholderia cepacia with phosphate solubilizing and a... |
| laurenceyang2018 | D2/D3 | Modeling the multi-scale mechanisms of macromolecular resource allocation |
| lolowalmarzan2011 | D1/D3 | Metabolic regulation of Escherichia coli and its phoB and phoR genes knockout mutants under phosphate and nitrogen limit... |
| manyou2020 | D3 | Isolation and characterization of Burkholderia cenocepacia CR318, a phosphate solubilizing bacterium promoting corn grow... |
| martindragosits2013 | D3 | Adaptive laboratory evolution – principles and applications for biotechnology |
| maríasoledadanzuay2015 | D3 | Beneficial effects of native phosphate solubilizing bacteria on peanut (Arachis hypogaea L) growth and phosphorus acquis... |
| maríasoledadanzuay2017 | D3 | Growth promotion of peanut ( Arachis hypogaea L.) and maize ( Zea mays L.) plants by single and mixed cultures of effici... |
| matthewrgoddard2003 | D3 | The adaptive response of a natural microbial population to carbon- and nitrogen-limitation |
| milkoajorquera2010 | D3 | Identification of β-propeller phytase-encoding genes in culturable Paenibacillus and Bacillus spp. from the rhizosphere ... |
| mivandyke2000 | D3 | Enhanced survival of Pseudomonas fluorescens in soil following establishment of inoculum in a sterile soil carrier |
| mmegharaj1997 | D3 | Superior survival and degradation of dibenzo-p-dioxin and dibenzofuran in soil by soil-adapted Sphingomonas sp. strain R... |
| muhammadshahid2012 | D3 | Root colonization and growth promotion of sunflower (Helianthus annuus L.) by phosphate solubilizing Enterobacter sp. Fs... |
| nabeelimam2021 | D3/D4 | Local Network Properties of Soil and Rhizosphere Microbial Communities in Potato Plantations Treated with a Biological P... |
| piaopiaochen2020 | D3 | Antagonistic pleiotropy conceals molecular adaptations in changing environments |
| polianaaparecidaleon2020 | D3/D4 | Inoculation With Growth-Promoting Bacteria Associated With the Reduction of Phosphate Fertilization in Sugarcane |
| qudsiasaeed2021 | D3 | Rhizosphere Bacteria in Plant Growth Promotion, Biocontrol, and Bioremediation of Contaminated Sites: A Comprehensive Re... |
| sabinearagot2016 | D1/D3 | Soil phoD and phoX alkaline phosphatase gene diversity responds to multiple environmental factors |
| sandratienda2024 | D3 | Development of a Pseudomonas -based biocontrol consortium with effective root colonization and extended beneficial side ... |
| sofiesnoeck2024 | D3 | “Metabolic burden” explained: stress symptoms and its related responses induced by (over)expression of (heterologous) pr... |
| yingxiaozhang2019 | D3/D4 | The emerging and uncultivated potential of CRISPR technology in plant science |
| yunpengliu2019 | D3 | Induced root-secreted d-galactose functions as a chemoattractant and enhances the biofilm formation of Bacillus velezens... |

## Table S2-4: Environmental Safety (Biocontainment and Evolutionary Stability) — Coded Papers: 68 (44.4% of total)

| Citation Key | Dims | Title |
| --- | --- | --- |
| aadishrawat2021 | D2/D4 | Cysteine proteases: Battling pathogenic parasitic protozoans with omnipresent enzymes |
| adrianaambrosini2015 | D3/D4 | Ecological role of bacterial inoculants and their potential impact on soil microbial diversity |
| ajaysingh2006 | D4 | Composting: A Potentially Safe Process for Disposal of Genetically Modified Organisms |
| alainajlapanse2022 | D3/D4 | Adaptive laboratory evolution for increased temperature tolerance of the diatom Nitzschia inconspicua |
| ambihaishayanthan2022 | D4 | The Role of Synthetic Microbial Communities (SynCom) in Sustainable Agriculture |
| binjia2017 | D4 | Orthogonal Ribosome Biofirewall |
| björnandreeßen2014 | D2/D3/D4 | Influence of the operon structure on poly(3-hydroxypropionate) synthesis in Shimwellia blattae |
| chengdongwang2023 | D4 | Phosphorus solubilizing microorganisms: potential promoters of agricultural and environmental engineering |
| christinenshulse2019 | D2/D4 | Engineered Root Bacteria Release Plant-Available Phosphate from Phytate |
| christophermwhitford2018 | D4 | Auxotrophy to Xeno-DNA: an exploration of combinatorial mechanisms for a high-fidelity biosafety system for synthetic bi... |
| claudiaelena2014 | D1/D2/D4 | Expression of codon optimized genes in microbial systems: current industrial applications and perspectives |
| collinrbarnum2021 | D4 | Utilizing Plant Synthetic Biology to Improve Human Health and Wellness |
| daltonrgeorge2024 | D4 | A bumpy road ahead for genetic biocontainment |
| danieljmandell2015 | D4 | Biocontainment of genetically modified organisms by synthetic protein design |
| daphnecollias2023 | D4 | Systematically attenuating DNA targeting enables CRISPR-driven editing in bacteria |
| deanpettinga2026 | D4 | Rational reduction of a sorghum SynCom that preserves growth promotion reveals flavonoid-mediated plant–microbe interact... |
| efsascientificcommit2020 | D4 | Evaluation of existing guidelines for their adequacy for the microbial characterisation and environmental risk assessmen... |
| emilkarlsen2018 | D4 | Automated generation of genome-scale metabolic draft reconstructions based on KEGG |
| esaúdelavegacamarill2023 | D3/D4 | Promotion of the growth and yield of Zea mays by synthetic microbial communities from Jala maize |
| feiranli2018 | D4 | Genome-scale metabolic model analysis indicates low energy production efficiency in marine ammonia-oxidizing archaea |
| fernandocarlosgómezm2015 | D4 | Plant and microbe genomics and beyond: potential for developing a novel molecular plant nutrition approach |
| fernandosantosbeneit2017 | D1/D4 | Glycopeptide resistance: Links with inorganic phosphate metabolism and cell envelope stress |
| finnstirling2017 | D4 | Rational Design of Evolutionarily Stable Microbial Kill Switches |
| finnstirling2020 | D4 | Synthetic Cassettes for pH-Mediated Sensing, Counting, and Containment |
| garywfoo2024 | D4 | Intein-based thermoregulated meganucleases for containment of genetic material |
| gmeyer2019 | D3/D4 | Pseudomonas protegens CHA0 does not increase phosphorus uptake from 33 P labeled synthetic hydroxyapatite by wheat grown... |
| graysonshamrick2024 | D4 | Programming Dynamic Division of Labor Using Horizontal Gene Transfer |
| jacquelinemchaparro2012 | D3/D4 | Manipulating the soil microbiome to increase soil health and plant fertility |
| jamesjbull2017 | D4 | Arresting Evolution |
| jingli2021 | D4 | Advances in Synthetic Biology and Biosafety Governance |
| jitendrawagh2014 | D1/D2/D4 | Heterologous expression of pyrroloquinoline quinone (pqq) gene cluster confers mineral phosphate solubilization ability ... |
| jitendrawagh2016 | D2/D4 | Inoculation of genetically modified endophytic Herbaspirillum seropedicae Z67 endowed with gluconic and 2-ketogluconic a... |
| juannogales2019 | D4 | High-quality genome-scale metabolic modelling of Pseudomonas putida highlights its broad metabolic capabilities |
| junhuang2022 | D2/D4 | CRISPR-Cas12a induced DNA double-strand breaks are repaired by multiple pathways with different mutation profiles in Mag... |
| kathleenlarnolds2021 | D4 | Biotechnology for secure biocontainment designs in an emerging bioeconomy |
| kmnielsen1997 | D4 | Evaluation of possible horizontal gene transfer from transgenic plants to the soil bacterium Acinetobacter calcoaceticus... |
| leipei2022 | D4 | Bottlenecks and opportunities for synthetic biology biosafety standards |
| liaobohua2023 | D4 | Ethical framework on risk governance of synthetic biology |
| marjorieahoy2000 | D4 | Transgenic Arthropods for Pest Management Programs: Risks and Realities |
| matanarbelgroissman2023 | D4 | Fighting the battle against evolution: designing genetically modified organisms for evolutionary stability |
| maureenocallaghan2024 | D4 | A synthetic microbial community used as a bioinoculant can overcome rice production constraints in acid soils |
| migmadorjitamang2014 | D4 | Characterization of plasmids encoding CTX-M β-lactamase and their addiction systems in Escherichia coli isolates from an... |
| mikemendelsohn2003 | D4 | Are Bt crops safe? |
| nabeelimam2021 | D3/D4 | Local Network Properties of Soil and Rhizosphere Microbial Communities in Potato Plantations Treated with a Biological P... |
| oliverwright2013 | D4 | Building-in biosafety for synthetic biology |
| olubukolaolurantibab2014 | D4 | Does nature make provision for backups in the modification of bacterial community structures? |
| panoslehouritis2017 | D4 | Designer bacteria as intratumoural enzyme biofactories |
| parizadbabaei2015 | D4 | Genome-scale reconstruction of the metabolic network in Pseudomonas stutzeri A1501 |
| peerapolchiaranunt2023 | D4 | Plant Beneficial Bacteria and Their Potential Applications in Vertical Farming Systems |
| polianaaparecidaleon2020 | D3/D4 | Inoculation With Growth-Promoting Bacteria Associated With the Reduction of Phosphate Fertilization in Sugarcane |
| randyortizcastro2019 | D4 | Review: Phytostimulation and root architectural responses to quorum-sensing signals and related molecules from rhizobact... |
| ryuichihirota2017 | D4 | A Novel Biocontainment Strategy Makes Bacterial Growth and Survival Dependent on Phosphite |
| sizheqiu2024 | D4 | DLTKcat: deep learning-based prediction of temperature-dependent enzyme turnover rates |
| stefanahoffmann2023i | D4 | Safety by design: Biosafety and biosecurity in the age of synthetic genomics |
| taeseokmoon2024 | D4 | Probiotic and microbiota engineering for practical applications |
| taosun2022 | D4 | Challenges and recent progress in the governance of biosecurity risks in the era of synthetic biology |
| tiffanymhalvorsen2022 | D4 | Comparison of Kill Switch Toxins in Plant-Beneficial Pseudomonas fluorescens Reveals Drivers of Lethality, Stability, an... |
| tingwang2026 | D4 | Synthetic microbial communities: from theoretical exploration to applied innovation |
| tinoflorybashizi2025 | D4 | Application of a Synthetic Microbial Community to Enhance Pepper Resistance Against Phytophthora capsici |
| wendycraig2008 | D4 | An overview of general features of risk assessments of genetically modified crops |
| wselbitschka1992 | D4 | The construction of recA–deficient Rhizobium meliloti and R. leguminosarum strains marked with gusA or luc cassettes for... |
| xiaoyanhuang2019 | D1/D4 | Reconstruction and analysis of a three-compartment genome-scale metabolic model for Pseudomonas fluorescens |
| yilou2024 | D4 | Biosafety and biosecurity: treading China's synbio tightrope |
| yingxiaozhang2019 | D3/D4 | The emerging and uncultivated potential of CRISPR technology in plant science |
| yuliang2022 | D4 | Construction of Environmental Synthetic Microbial Consortia: Based on Engineering and Ecological Principles |
| yunhecui2025 | D4 | Harnessing synthetic microbial communities for sustainable agriculture: Enhancing soil health and crop yields |
| yusukekato2024 | D4 | Genetically stable kill-switch using “demon and angel” expression construct of essential genes |
| zhiminyang2018 | D4 | Global investigation of an engineered nitrogen-fixing Escherichia coli strain reveals regulatory coupling between host a... |

## Table S3: Complete Coded Literature Inventory (153 coded + 10 uncoded)

| Citation Key | Code | Title | Year |
| --- | --- | --- | --- |
| aadishrawat2021 | D2/D4 | Cysteine proteases: Battling pathogenic parasitic protozoans with omnipresent enzymes | 2021 |
| aditibuch2010 | D2 | Heterologous expression of phosphoenolpyruvate carboxylase enhances the phosphate solubilizing ability of fluorescent ps... | 2009 |
| adrianaambrosini2015 | D3/D4 | Ecological role of bacterial inoculants and their potential impact on soil microbial diversity | 2015 |
| ajaysingh2006 | D4 | Composting: A Potentially Safe Process for Disposal of Genetically Modified Organisms | 2006 |
| alainajlapanse2022 | D3/D4 | Adaptive laboratory evolution for increased temperature tolerance of the diatom Nitzschia inconspicua | 2022 |
| alexanderdasilvavale2022 | D2 | Perspective on the use of synthetic biology in rudimentary food fermentations | 2022 |
| allisonsharrar2024 | D2 | Discovery and engineering of AiEvo2, a novel Cas12a nuclease for human gene editing applications | 2024 |
| amandineducoussodétr2022 | D3 | Diversity of Phosphate Chemical Forms in Soils and Their Contributions on Soil Microbial Community Structure Changes | 2022 |
| ambihaishayanthan2022 | D4 | The Role of Synthetic Microbial Communities (SynCom) in Sustainable Agriculture | 2022 |
| andrewlneal2017 | D1 | Phylogenetic distribution, biogeography and the effects of land management upon bacterial non-specific Acid phosphatase ... | 2017 |
| annatimofeeva2022 | D3 | Prospects for Using Phosphate-Solubilizing Microorganisms as Natural Fertilizers in Agriculture | 2022 |
| asfarizvi2021 | D3 | Psychrophilic Bacterial Phosphate-Biofertilizers: A Novel Extremophile for Sustainable Crop Production under Cold Enviro... | 2021 |
| benjesuorsemwenenagb2020 | D1 | Unveiling Plant-Beneficial Function as Seen in Bacteria Genes from Termite Mound Soil | 2020 |
| bijoyapaul2020 | D2 | CRISPR-Cas12a: Functional overview and applications | 2020 |
| binjia2017 | D4 | Orthogonal Ribosome Biofirewall | 2017 |
| birgitescharf2016 | D3 | Chemotaxis signaling systems in model beneficial plant–bacteria associations | 2016 |
| björnandreeßen2014 | D2/D3/D4 | Influence of the operon structure on poly(3-hydroxypropionate) synthesis in Shimwellia blattae | 2014 |
| brianarenda2014 | D3 | Genome Instability Mediates the Loss of Key Traits by Acinetobacter baylyi ADP1 during Laboratory Evolution | 2014 |
| chengdongwang2023 | D4 | Phosphorus solubilizing microorganisms: potential promoters of agricultural and environmental engineering | 2023 |
| christinenshulse2019 | D2/D4 | Engineered Root Bacteria Release Plant-Available Phosphate from Phytate | 2019 |
| christophermwhitford2018 | D4 | Auxotrophy to Xeno-DNA: an exploration of combinatorial mechanisms for a high-fidelity biosafety system for synthetic bi... | 2018 |
| claudiaelena2014 | D1/D2/D4 | Expression of codon optimized genes in microbial systems: current industrial applications and perspectives | 2014 |
| collinrbarnum2021 | D4 | Utilizing Plant Synthetic Biology to Improve Human Health and Wellness | 2021 |
| daltonrgeorge2024 | D4 | A bumpy road ahead for genetic biocontainment | 2024 |
| danieljmandell2015 | D4 | Biocontainment of genetically modified organisms by synthetic protein design | 2015 |
| daphnecollias2023 | D4 | Systematically attenuating DNA targeting enables CRISPR-driven editing in bacteria | 2023 |
| deanpettinga2026 | D4 | Rational reduction of a sorghum SynCom that preserves growth promotion reveals flavonoid-mediated plant–microbe interact... | 2026 |
| dehongzheng2018 | D1 | Activation of PhoBR under phosphate-rich conditions reduces the virulence of Xanthomonas oryzae pv. oryzae | 2018 |
| deniselüttmann2012 | D1 | The phosphotransferase protein EIIANtr modulates the phosphate starvation response through interaction with histidine ki... | 2012 |
| dmitryderyabin2019 | D3 | Plant-Derived Inhibitors of AHL-Mediated Quorum Sensing in Bacteria: Modes of Action | 2019 |
| dorothébelotte2007 | D3 | An experimental test of local adaptation in soil bacteria | 2003 |
| efsascientificcommit2020 | D4 | Evaluation of existing guidelines for their adequacy for the microbial characterisation and environmental risk assessmen... | 2020 |
| emilkarlsen2018 | D4 | Automated generation of genome-scale metabolic draft reconstructions based on KEGG | 2018 |
| esaúdelavegacamarill2023 | D3/D4 | Promotion of the growth and yield of Zea mays by synthetic microbial communities from Jala maize | 2023 |
| esmaeilbakhshandeh2014 | D3 | Phosphate solubilization potential and modeling of stress tolerance of rhizobacteria from rice paddy soil in northern Ir... | 2014 |
| estherblancoromero2023 | D3 | Adaption of Pseudomonas ogarae F113 to the Rhizosphere Environment—The AmrZ-FleQ Hub | 2023 |
| feiranli2018 | D4 | Genome-scale metabolic model analysis indicates low energy production efficiency in marine ammonia-oxidizing archaea | 2018 |
| fernandocarlosgómezm2015 | D4 | Plant and microbe genomics and beyond: potential for developing a novel molecular plant nutrition approach | 2015 |
| fernandosantosbeneit2017 | D1/D4 | Glycopeptide resistance: Links with inorganic phosphate metabolism and cell envelope stress | 2016 |
| finnstirling2017 | D4 | Rational Design of Evolutionarily Stable Microbial Kill Switches | 2017 |
| finnstirling2020 | D4 | Synthetic Cassettes for pH-Mediated Sensing, Counting, and Containment | 2020 |
| franciscoxnascimento2021 | D1 | Genomic Analysis of the 1-Aminocyclopropane-1-Carboxylate Deaminase-Producing Pseudomonas thivervalensis SC5 Reveals Its... | 2021 |
| fujingpan2024 | D1 | Vegetation recovery reshapes the composition and enhances the network connectivity of phoD -harboring microorganisms to ... | 2024 |
| garywfoo2024 | D4 | Intein-based thermoregulated meganucleases for containment of genetic material | 2024 |
| gmeyer2019 | D3/D4 | Pseudomonas protegens CHA0 does not increase phosphorus uptake from 33 P labeled synthetic hydroxyapatite by wheat grown... | 2019 |
| graysonshamrick2024 | D4 | Programming Dynamic Division of Labor Using Horizontal Gene Transfer | 2024 |
| guillermofernandezbu2022 | D2/D3 | Novel Production Methods of Polyhydroxyalkanoates and Their Innovative Uses in Biomedicine and Industry | 2022 |
| gustavosantoyo2021 | D3 | Rhizosphere Colonization Determinants by Plant Growth-Promoting Rhizobacteria (PGPR) | 2021 |
| huiwan2017 | D1 | Identification of transporter proteins for PQQ-secretion pathways by transcriptomics and proteomics analysis in Gluconob... | 2016 |
| huoqinghuang2009 | D1 | Diversity of Beta-Propeller Phytase Genes in the Intestinal Contents of Grass Carp Provides Insight into the Release of ... | 2009 |
| hyadav2013 | D3 | Optimization of culture conditions for phosphate solubilization by a thermo-tolerant phosphate-solubilizing bacterium Br... | 2013 |
| idaromano2020 | D3 | Effectiveness of Plant Beneficial Microbes: Overview of the Methodological Approaches for the Assessment of Root Coloniz... | 2020 |
| igordanielalvesribei2020 | D2 | Use of Mineral Weathering Bacteria to Enhance Nutrient Availability in Crops: A Review | 2020 |
| ilhemsaadouli2021 | D1 | The Impact of the Inoculation of Phosphate-Solubilizing Bacteria Pantoea agglomerans on Phosphorus Availability and Bact... | 2021 |
| israelikoyi2024 | D3 | Manipulation of sward diversity is a more effective management strategy than addition of microbial inoculants in intensi... | 2024 |
| ivanmijakovic2005 | D2 | Tunable promoters in systems biology | 2005 |
| jacquelinemchaparro2012 | D3/D4 | Manipulating the soil microbiome to increase soil health and plant fertility | 2012 |
| jamesjbull2017 | D4 | Arresting Evolution | 2017 |
| jessicaaparecidaferr2023 | D3 | Meta-omics integration approach reveals the effect of soil native microbiome diversity in the performance of inoculant A... | 2023 |
| jianli2021 | D2 | Microbial-Derived Polyhydroxyalkanoate-Based Scaffolds for Bone Tissue Engineering: Biosynthesis, Properties, and Perspe... | 2021 |
| jingli2021 | D4 | Advances in Synthetic Biology and Biosafety Governance | 2021 |
| jitendrawagh2014 | D1/D2/D4 | Heterologous expression of pyrroloquinoline quinone (pqq) gene cluster confers mineral phosphate solubilization ability ... | 2014 |
| jitendrawagh2016 | D2/D4 | Inoculation of genetically modified endophytic Herbaspirillum seropedicae Z67 endowed with gluconic and 2-ketogluconic a... | 2016 |
| joshuarelmore2023 | D2 | High-throughput genetic engineering of nonmodel and undomesticated bacteria via iterative site-specific genome integrati... | 2023 |
| juannogales2019 | D4 | High-quality genome-scale metabolic modelling of Pseudomonas putida highlights its broad metabolic capabilities | 2019 |
| junhuang2022 | D2/D4 | CRISPR-Cas12a induced DNA double-strand breaks are repaired by multiple pathways with different mutation profiles in Mag... | 2022 |
| justintan2020 | D2/D3 | Independent component analysis of E. coli 's transcriptome reveals the cellular processes that respond to heterologous g... | 2020 |
| kathleenlarnolds2021 | D4 | Biotechnology for secure biocontainment designs in an emerging bioeconomy | 2021 |
| katilavarivarn2013 | D3 | Colonization strategies of Pseudomonas fluorescens Pf0-1: activation of soil-specific genes important for diverse and sp... | 2013 |
| kellybotero2018 | D1 | A genome-scale metabolic model of potato late blight suggests a photosynthesis suppression mechanism | 2018 |
| kezhao2014 | D3 | Maize rhizosphere in Sichuan, China, hosts plant growth promoting Burkholderia cepacia with phosphate solubilizing and a... | 2013 |
| kmnielsen1997 | D4 | Evaluation of possible horizontal gene transfer from transgenic plants to the soil bacterium Acinetobacter calcoaceticus... | 1997 |
| laurenceyang2018 | D2/D3 | Modeling the multi-scale mechanisms of macromolecular resource allocation | 2018 |
| leipei2022 | D4 | Bottlenecks and opportunities for synthetic biology biosafety standards | 2022 |
| liaobohua2023 | D4 | Ethical framework on risk governance of synthetic biology | 2023 |
| licunzhang2023 | D1 | Land reclamation increased organic P fractions and phosphatase activities, and strengthened the co-occurrence networks o... | 2023 |
| linlinwang2022 | D1 | Genomic Analysis of Pseudomonas asiatica JP233: An Efficient Phosphate-Solubilizing Bacterium | 2022 |
| liqunjin2019 | D2 | Promoter engineering strategies for the overproduction of valuable metabolites in microbes | 2019 |
| lolowalmarzan2011 | D1/D3 | Metabolic regulation of Escherichia coli and its phoB and phoR genes knockout mutants under phosphate and nitrogen limit... | 2011 |
| manyou2020 | D3 | Isolation and characterization of Burkholderia cenocepacia CR318, a phosphate solubilizing bacterium promoting corn grow... | 2019 |
| marjorieahoy2000 | D4 | Transgenic Arthropods for Pest Management Programs: Risks and Realities | 2000 |
| martindragosits2013 | D3 | Adaptive laboratory evolution – principles and applications for biotechnology | 2013 |
| maríasoledadanzuay2015 | D3 | Beneficial effects of native phosphate solubilizing bacteria on peanut (Arachis hypogaea L) growth and phosphorus acquis... | 2015 |
| maríasoledadanzuay2017 | D3 | Growth promotion of peanut ( Arachis hypogaea L.) and maize ( Zea mays L.) plants by single and mixed cultures of effici... | 2017 |
| matanarbelgroissman2023 | D4 | Fighting the battle against evolution: designing genetically modified organisms for evolutionary stability | 2023 |
| matthewrgoddard2003 | D3 | The adaptive response of a natural microbial population to carbon- and nitrogen-limitation | 2003 |
| maureenocallaghan2024 | D4 | A synthetic microbial community used as a bioinoculant can overcome rice production constraints in acid soils | 2024 |
| meiyiyan2017 | D2 | CRISPR-Cas12a-Assisted Recombineering in Bacteria | 2017 |
| meliawatimeliawati2021 | D2 | Recent advances of Cas12a applications in bacteria | 2021 |
| michellejchua2022 | D2 | Rapid, Efficient, and Cost-Effective Gene Editing of Enterococcus faecium with CRISPR-Cas12a | 2022 |
| migmadorjitamang2014 | D4 | Characterization of plasmids encoding CTX-M β-lactamase and their addiction systems in Escherichia coli isolates from an... | 2014 |
| mikemendelsohn2003 | D4 | Are Bt crops safe? | 2003 |
| milkoajorquera2010 | D3 | Identification of β-propeller phytase-encoding genes in culturable Paenibacillus and Bacillus spp. from the rhizosphere ... | 2010 |
| mingzhao2019 | D2 | Engineering diverse eubacteria promoters for robust Gene expression in Streptomyces lividans | 2018 |
| mivandyke2000 | D3 | Enhanced survival of Pseudomonas fluorescens in soil following establishment of inoculum in a sterile soil carrier | 2000 |
| mmegharaj1997 | D3 | Superior survival and degradation of dibenzo-p-dioxin and dibenzofuran in soil by soil-adapted Sphingomonas sp. strain R... | 1997 |
| monisatharkhan2026 | D2 | Targeted genome editing of the non-model cyanobacterium Cyanothece PCC 7425 via CRISPR/Cas12a | 2026 |
| muhammadkashifhanif2015 | D1 | Isolation and characterization of a β-propeller gene containing phosphobacterium Bacillus subtilis strain KPS-11 for gro... | 2015 |
| muhammadshahid2012 | D3 | Root colonization and growth promotion of sunflower (Helianthus annuus L.) by phosphate solubilizing Enterobacter sp. Fs... | 2012 |
| nabeelimam2021 | D3/D4 | Local Network Properties of Soil and Rhizosphere Microbial Communities in Potato Plantations Treated with a Biological P... | 2021 |
| nataliastosiek2019 | D1 | Carbon-Phosphorus Lyase—the State of the Art | 2019 |
| ningxu2019 | D2 | Recent advances in the applications of promoter engineering for the optimization of metabolite biosynthesis | 2019 |
| oliverwright2013 | D4 | Building-in biosafety for synthetic biology | 2013 |
| olubukolaolurantibab2014 | D4 | Does nature make provision for backups in the modification of bacterial community structures? | 2014 |
| panoslehouritis2017 | D4 | Designer bacteria as intratumoural enzyme biofactories | 2017 |
| parizadbabaei2015 | D4 | Genome-scale reconstruction of the metabolic network in Pseudomonas stutzeri A1501 | 2015 |
| peerapolchiaranunt2023 | D4 | Plant Beneficial Bacteria and Their Potential Applications in Vertical Farming Systems | 2023 |
| peichunlisahsu2015 | D1 | hemX is required for production of 2‐ketogluconate, the predominant organic anion required for inorganic phosphate solub... | 2015 |
| piaopiaochen2020 | D3 | Antagonistic pleiotropy conceals molecular adaptations in changing environments | 2020 |
| polianaaparecidaleon2020 | D3/D4 | Inoculation With Growth-Promoting Bacteria Associated With the Reduction of Phosphate Fertilization in Sugarcane | 2020 |
| prassanchoudhary2022 | D1 | Understanding the biological role of PqqB in Pseudomonas stutzeri using molecular dynamics simulation approach | 2020 |
| qudsiasaeed2021 | D3 | Rhizosphere Bacteria in Plant Growth Promotion, Biocontrol, and Bioremediation of Contaminated Sites: A Comprehensive Re... | 2021 |
| rajinderkaur2021 | D1 | Molecular and physiological manipulations in rhizospheric bacteria | 2021 |
| ranan2016 | D1 | Regulation of Pyrroloquinoline Quinone-Dependent Glucose Dehydrogenase Activity in the Model Rhizosphere-Dwelling Bacter... | 2016 |
| randyortizcastro2019 | D4 | Review: Phytostimulation and root architectural responses to quorum-sensing signals and related molecules from rhizobact... | 2019 |
| ryuichihirota2017 | D4 | A Novel Biocontainment Strategy Makes Bacterial Growth and Survival Dependent on Phosphite | 2017 |
| sabinearagot2015 | D1 | phoD Alkaline Phosphatase Gene Diversity in Soil | 2015 |
| sabinearagot2016 | D1/D3 | Soil phoD and phoX alkaline phosphatase gene diversity responds to multiple environmental factors | 2016 |
| samikshajoshi2023 | D1 | Functional characterization and molecular fingerprinting of potential phosphate solubilizing bacterial candidates from S... | 2023 |
| sandratienda2024 | D3 | Development of a Pseudomonas -based biocontrol consortium with effective root colonization and extended beneficial side ... | 2024 |
| sarabaldanta2022 | D2 | SEVA-Cpf1, a CRISPR-Cas12a vector for genome editing in cyanobacteria | 2022 |
| scaria2010 | D1 | Crystallization and preliminary X-ray characterization of a catalytic and ATP-binding domain of a putative PhoR histidin... | 2010 |
| sizheqiu2024 | D4 | DLTKcat: deep learning-based prediction of temperature-dependent enzyme turnover rates | 2023 |
| sofiesnoeck2024 | D3 | “Metabolic burden” explained: stress symptoms and its related responses induced by (over)expression of (heterologous) pr... | 2024 |
| stefanahoffmann2023i | D4 | Safety by design: Biosafety and biosecurity in the age of synthetic genomics | 2023 |
| taeseokmoon2024 | D4 | Probiotic and microbiota engineering for practical applications | 2024 |
| tandradfraser2015 | D1 | Soil bacterial phoD gene abundance and expression in response to applied phosphorus and long-term management | 2015 |
| taosun2022 | D4 | Challenges and recent progress in the governance of biosecurity risks in the era of synthetic biology | 2022 |
| tiffanymhalvorsen2022 | D4 | Comparison of Kill Switch Toxins in Plant-Beneficial Pseudomonas fluorescens Reveals Drivers of Lethality, Stability, an... | 2022 |
| tingwang2026 | D4 | Synthetic microbial communities: from theoretical exploration to applied innovation | 2026 |
| tinoflorybashizi2025 | D4 | Application of a Synthetic Microbial Community to Enhance Pepper Resistance Against Phytophthora capsici | 2025 |
| weimeng2023 | D2 | Construction and application of a new CRISPR/Cas12a system in Stenotrophomonas AGS-1 from aerobic granular sludge | 2023 |
| wendycraig2008 | D4 | An overview of general features of risk assessments of genetically modified crops | 2008 |
| wenjiewan2020 | D1 | Isolation and Characterization of Phosphorus Solubilizing Bacteria With Multiple Phosphorus Sources Utilizing Capability... | 2020 |
| wenzhu2020 | D1 | Biogenesis of the peptide-derived redox cofactor pyrroloquinoline quinone | 2020 |
| wselbitschka1992 | D4 | The construction of recA–deficient Rhizobium meliloti and R. leguminosarum strains marked with gusA or luc cassettes for... | 1992 |
| xiangao2018 | D2 | A Multiplex Genome Editing Method for Escherichia coli Based on CRISPR-Cas12a | 2018 |
| xiaodongchen2021 | D1 | Impact of grassland afforestation with contrasting tree species on soil phosphorus fractions and alkaline phosphatase ge... | 2021 |
| xiaofanfeng2021 | D2 | Saccharomyces cerevisiae Promoter Engineering before and during the Synthetic Biology Era | 2021 |
| xiaoyanhuang2019 | D1/D4 | Reconstruction and analysis of a three-compartment genome-scale metabolic model for Pseudomonas fluorescens | 2019 |
| xuemihao2023 | D2 | CRISPR/Cas12a-Mediated Genome Editing in Thioalkalivibrio versutus | 2023 |
| xuzhang2020 | D2 | Synthetic Biology and Genome-Editing Tools for Improving PHA Metabolic Engineering | 2019 |
| yajunhu2018 | D1 | Effects of long-term fertilization on phoD -harboring bacterial community in Karst soils | 2018 |
| yaoqingshen2012 | D1 | Distribution and Properties of the Genes Encoding the Biosynthesis of the Bacterial Cofactor, Pyrroloquinoline Quinone | 2012 |
| yilou2024 | D4 | Biosafety and biosecurity: treading China's synbio tightrope | 2023 |
| yingxiaozhang2019 | D3/D4 | The emerging and uncultivated potential of CRISPR technology in plant science | 2019 |
| yuhuizhang2018 | D1 | GlnR positive transcriptional regulation of the phosphate-specific transport system pstSCAB in Amycolatopsis mediterrane... | 2018 |
| yujiezhang2025 | D2 | Establishment of a RecET-Assisted CRISPR-Cas12a System for Large Deoxyribonucleic Acid-Fragment Manipulation in Zymomona... | 2025 |
| yuliang2022 | D4 | Construction of Environmental Synthetic Microbial Consortia: Based on Engineering and Ecological Principles | 2022 |
| yunhecui2025 | D4 | Harnessing synthetic microbial communities for sustainable agriculture: Enhancing soil health and crop yields | 2025 |
| yunpengliu2019 | D3 | Induced root-secreted d-galactose functions as a chemoattractant and enhances the biofilm formation of Bacillus velezens... | 2019 |
| yusukekato2024 | D4 | Genetically stable kill-switch using “demon and angel” expression construct of essential genes | 2024 |
| zhiminyang2018 | D4 | Global investigation of an engineered nitrogen-fixing Escherichia coli strain reveals regulatory coupling between host a... | 2018 |
| avishekshaw2022 | --- | A Review on the Role of Phosphate Solubilizing Microorganisms with a Brief Focus on its Genetic Aspects. | 2022 |
| bcbehera2014 | --- | Diversity, mechanism and biotechnology of phosphate solubilising microorganism in mangrove—A review | 2013 |
| carlosfernandogutiér2022 | --- | Exploiting the Potential of Bioreactors for Creating Spatial Organization in the Soil Microbiome: A Strategy for Increas... | 2022 |
| ffatima2021 | --- | Relevance of phosphate solubilizing microbes in sustainable crop production: a review | 2021 |
| karlagabrieladomíngu2022 | --- | Streptomyces spp. Biofilmed Solid Inoculant Improves Microbial Survival and Plant-Growth Efficiency of Triticum aestivum | 2022 |
| mariarasul2019 | --- | Glucose dehydrogenase gene containing phosphobacteria for biofortification of Phosphorus with growth promotion of rice | 2019 |
| rifathayat2010 | --- | Soil beneficial bacteria and their role in plant growth promotion: a review | 2010 |
| sannewmpoppeliers2023 | --- | Microbes to support plant health: understanding bioinoculant success in complex conditions | 2023 |
| toshimimichigami2018 | --- | Phosphate as a Signaling Molecule and Its Sensing Mechanism | 2018 |
| xuechaoqin2020 | --- | How long-term excessive manure application affects soil phosphorous species and risk of phosphorous loss in fluvo-aquic ... | 2020 |
